# BiP Binding to the ER-Stress Sensor Ire1 Tunes the Homeostatic Behavior of the Unfolded Protein Response

**DOI:** 10.1371/journal.pbio.1000415

**Published:** 2010-07-06

**Authors:** David Pincus, Michael W. Chevalier, Tomás Aragón, Eelco van Anken, Simon E. Vidal, Hana El-Samad, Peter Walter

**Affiliations:** 1 Department of Biochemistry and Biophysics, University of California at San Francisco, San Francisco, California, United States of America; 2 Howard Hughes Medical Institute, University of California at San Francisco, San Francisco, California, Unites States of America; Scripps Research Institute, United States of America

## Abstract

The unfolded protein response (UPR) is an intracellular signaling pathway that counteracts variable stresses that impair protein folding in the endoplasmic reticulum (ER). As such, the UPR is thought to be a homeostat that finely tunes ER protein folding capacity and ER abundance according to need. The mechanism by which the ER stress sensor Ire1 is activated by unfolded proteins and the role that the ER chaperone protein BiP plays in Ire1 regulation have remained unclear. Here we show that the UPR matches its output to the magnitude of the stress by regulating the duration of Ire1 signaling. BiP binding to Ire1 serves to desensitize Ire1 to low levels of stress and promotes its deactivation when favorable folding conditions are restored to the ER. We propose that, mechanistically, BiP achieves these functions by sequestering inactive Ire1 molecules, thereby providing a barrier to oligomerization and activation, and a stabilizing interaction that facilitates de-oligomerization and deactivation. Thus BiP binding to or release from Ire1 is not instrumental for switching the UPR on and off as previously posed. By contrast, BiP provides a buffer for inactive Ire1 molecules that ensures an appropriate response to restore protein folding homeostasis to the ER by modulating the sensitivity and dynamics of Ire1 activity.

## Abstract 2

Computational modeling and experimentation in the unfolded protein response reveals a role for the ER-resident chaperone protein BiP in fine-tuning the system's response dynamics.

## Author Summary

Secreted and membrane-spanning proteins constitute one of every three proteins produced by a eukaryotic cell. Many of these proteins initially fold and assemble in the endoplasmic reticulum (ER). A variety of physiological and environmental conditions can increase the demands on the ER, overwhelming the ER protein folding machinery. To restore homeostasis in response to ER stress, cells activate an intracellular signaling pathway called the unfolded protein response (UPR) that adjusts the folding capacity of the ER according to need. Its failure impairs cell viability and has been implicated in numerous disease states. In this study, we quantitatively interrogate the homeostatic capacity of the UPR. We arrive at a mechanistic model for how the ER stress sensor Ire1 cooperates with its binding partner BiP, a highly redundant ER chaperone, to fine-tune UPR activity. Moving between a predictive computational model and experiments, we show that BiP release from Ire1 is not the switch that activates Ire1; rather, BiP modulates Ire1 activation and deactivation dynamics. BiP binding to Ire1 and its dissociation in an ER stress-dependent manner buffers the system against mild stresses. Furthermore, BiP binding accelerates Ire1 deactivation when stress is removed. We conclude that BiP binding to Ire1 serves to fine-tune the dynamic behavior of the UPR by modulating its sensitivity and shutoff kinetics. This function of the interaction between Ire1 and BiP may be a general paradigm for other systems in which oligomer formation and disassembly must be finely regulated.

## Introduction

The secreted and membrane-spanning proteins that eukaryotic cells use to sense and respond to their environments and to communicate with other cells are functional only when they attain their proper three-dimensional structures. Folding of these proteins takes place in the endoplasmic reticulum (ER), aided by molecular chaperones. Degradation pathways help to discard misfolded proteins. When cells experience environmental stresses, nutrient depletion, or certain differentiation cues, the ER folding and degradation machineries can become overwhelmed and the cell risks accumulating and secreting malfunctional and potentially harmful proteins [1]. Such conditions of ER stress activate the unfolded protein response (UPR) [2], resulting in an expanded ER [3],[4] and increased expression of genes encoding ER chaperones, ER associated degradation machinery, and other components of the secretory pathway [5]. As such, the UPR provides a feedback loop that helps cells maintain high fidelity in protein folding and assembly.

The UPR plays a fundamental role in maintaining cellular homeostasis and is therefore at the center of many normal physiological responses and pathologies. For example, when the severity of ER stress exceeds the capacity of the UPR to restore homeostasis, mammalian cells commit to apoptosis [2]. Furthermore, the UPR is activated in many cancer cells [6],[7],[8] as well as during familial protein-folding and neurodegenerative diseases [9],[10]. Deficiencies in UPR signaling can also lead to diabetes [11]. Thus, the UPR constitutes an important control module whose core signaling machinery, which is conserved from yeast to humans, proves critical for cell physiology.

Misfolded secretory proteins accumulate in the ER lumen. The UPR is initiated in that compartment when the transmembrane sensor molecule Ire1 self-associates and activates its cytoplasmic endoribonuclease domain [12],[13],[14],[15]. Activated Ire1 transmits the signal by removing a non-conventional intron from its mRNA substrates, *HAC1* mRNA in yeast and XBP1 mRNA in metazoans, which upon subsequent ligation are translated to produce potent transcriptional activators of UPR target genes [16],[17],[18]. Since the Hac1 protein is short-lived (half-life of ∼2 min) [18],[19], Ire1 activity is the key determinant of the magnitude and duration of the UPR.

Despite early clues for Ire1's role as a central UPR regulator, the mechanism by which it senses unfolded proteins remains disputed. One model proposes that Ire1 activity is mainly regulated by the ER-resident chaperone BiP (Kar2 in yeast). In this model, BiP inhibits Ire1 activity by binding to it in the absence of stress. During stress, BiP is titrated away by unfolded proteins, leaving Ire1 free to oligomerize and activate. This model was suggested because immunoprecipitation experiments showed that Ire1 interacts with BiP in unstressed cells and dissociates from BiP under ER stress conditions [20],[21],[22]. Site directed mutagenesis of BiP yielded mutants that do not bind to Ire1 [23], but since they failed to support growth when expressed as the only copy of BiP, they are difficult to interpret mechanistically in view of the many pleiotropic functions of BiP. By contrast, mutants of Ire1 lacking the juxtamembrane segment of its lumenal domain that is responsible for BiP binding retained regulation: mutant Ire1 was inactive in the absence of ER stress and activated in its presence [15],[22],[24],[25], thus suggesting that BiP release and rebinding are not causal for switching Ire1 on and off.

An alternative model of Ire1 regulation postulates that unfolded proteins bind to the lumenal domain of Ire1, triggering Ire1 self-association and activation of its cytoplasmic effector domains. Support for such activation of Ire1 by direct binding to unfolded proteins stems from structural studies of the Ire1 lumenal domain that revealed a putative peptide binding groove [24]. Mutational probing experiments demonstrated that the residues pointing into the groove are required for signaling [24].

Recently a hybrid, two-step model for UPR regulation has been proposed in which both BiP and unfolded proteins regulate Ire1: initial dissociation of BiP from Ire1 drives its oligomerization, while subsequent binding to unfolded proteins leads to its activation [15]. This model posits that BiP regulates Ire1 oligomerization, yet oligomerization is not sufficient for Ire1 activation. However, in vitro experiments demonstrated that the oligomerization state of the cytoplasmic domains of Ire1 determines the rate of enzymatic activity [12].

Thus, while genetic and biochemical analyses of the UPR have been immensely successful in elucidating many aspects of the UPR's unusual signal transduction mechanism, a coherent model of Ire1 regulation and the involvement of BiP has remained elusive. In this work, we study the UPR as a coordinated homeostatic system by carrying out measurements of the time dynamics of the pathway across a wide range of ER stress levels. Using population-based assays of UPR activity complemented with dynamic dose-resolved flow cytometry and a predictive computational model, we dissect the role of BiP in modulating the sensitivity and duration of the UPR. Specifically, by comparing the wild type UPR to a strain bearing a mutant version of Ire1 that lacks the UPR-specific BiP interaction motif, we show that BiP prevents Ire1 from activating in response to low levels of stress and that it aids in Ire1 deactivation once the stress has been alleviated. Using a single cell Ire1 FRET assay, we provide evidence suggesting that BiP performs these functions by sequestering inactive Ire1 molecules. By buffering Ire1, BiP ensures that only appropriate levels of stress trigger the UPR and that the duration of UPR induction matches the magnitude of the stress. These data position BiP as a modulator of the dynamic properties of the UPR.

## Results

### The Unfolded Protein Sensor Ire1 Undergoes Activation and Deactivation

**Figure 1:**
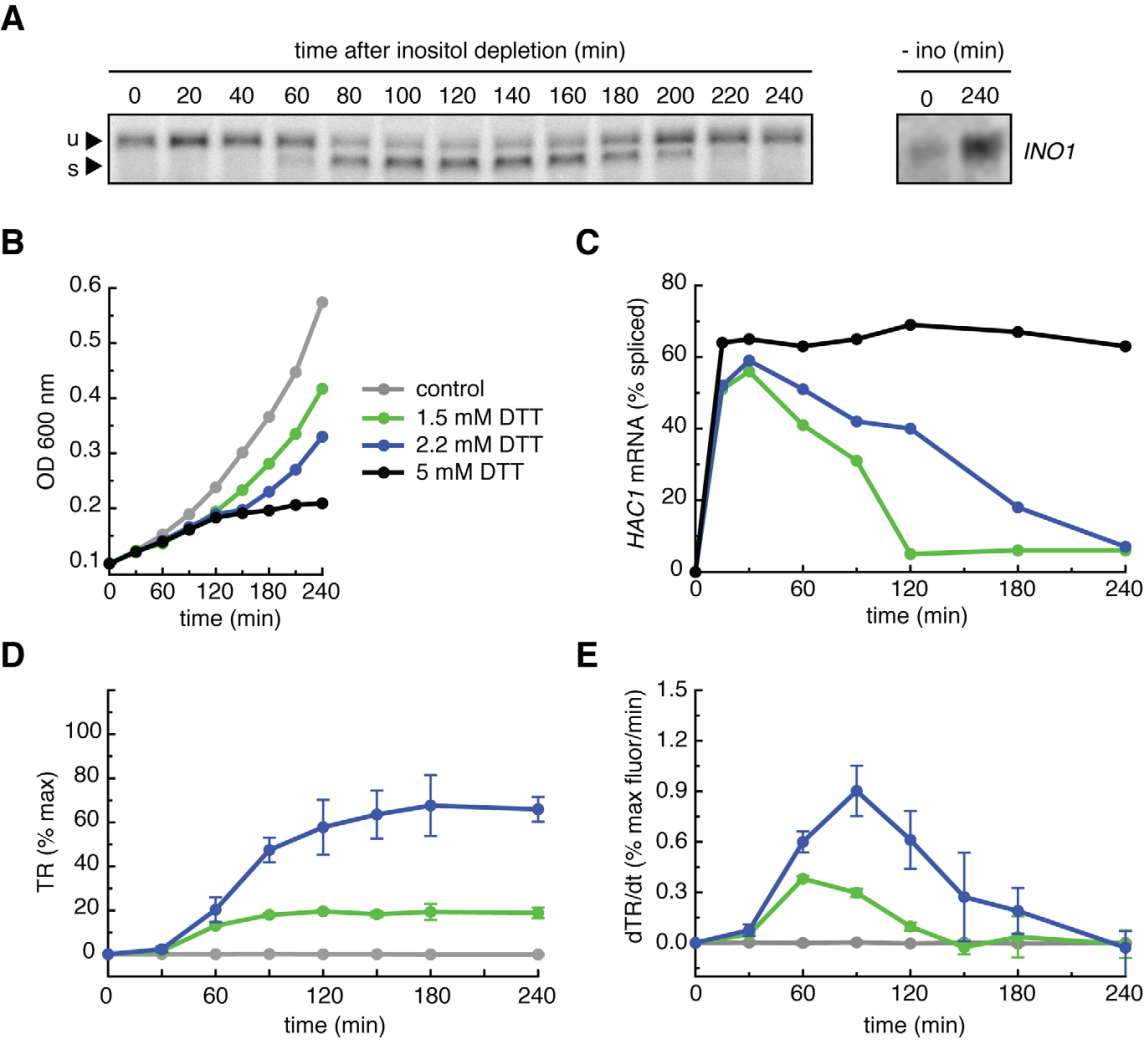
Transient Ire1 activation in non-lethal ER stress conditions. (A) After depletion of inositol from the growth media, wild type yeast cells were sampled from a master culture every 20 min, and total RNA was purified and subjected to Northern blot analysis using a probe for the first exon of *HAC1* mRNA. After a lag phase, *HAC1* mRNA splicing displayed activation and deactivation phases. u, unspliced *HAC1* mRNA; s, spliced *HAC1* mRNA. Right panel: wild type cells 0 min and 240 min after inositol depletion and probed for the *INO1* mRNA. (B) Cell growth was monitored over time in wild type cells treated with 5 mM, 2.2 mM, 1.5 mM, and 0 mM DTT by measuring the OD_600_. Cells treated with 5 mM DTT cease to divide, while cells treated with 2.2 mM or 1.5 mM DTT continue to grow. (C) Wild type cells were treated with 5 mM, 2.2 mM, or 1.5 mM DTT and sampled over time. After Northern blot analysis, the percentage of spliced *HAC1* mRNA was quantified (blots are shown in the supplement). Cells treated with 5 mM DTT displayed sustained maximal splicing, while cells treated with 2.2 mM or 1.5 mM displayed transient *HAC1* mRNA splicing: the same activation and deactivation phases as the response to the depletion of inositol. (D) Wild type cells were constructed bearing a transcriptional reporter (TR) consisting of four repeats of a UPR-responsive DNA element controlling the expression of GFP. These cells were treated with 2.2 mM, 1.5 mM, or 0 mM DTT, sampled over time, and subjected to flow cytometry to quantify the GFP fluorescence. The TR was induced to dose-dependent plateaus due to the >8 h half life of GFP. % max is defined as the GFP fluorescence in cells treated with 5 mM DTT for 4 h. (E) When plotted as the rate of GFP produced per minute, the TR displayed the same activation and deactivation phases as spliced *HAC1* mRNA. Transient Ire1 activation leads to transient transcriptional activation. % max as defined in (D).

Most UPR studies to date have been carried out under saturating conditions, where induction of protein folding damage surpasses the homeostatic capacity of the UPR and hence remains unmitigated. To position the experimental system in a physiological regime where cells proliferate efficiently when the UPR functions adequately, we probed the response to depletion of the metabolite inositol [26]. In the absence of inositol in the growth media, Ire1 is required for cells to induce the expression of genes required for inositol synthesis as part of the UPR transcriptional program [27]. To monitor UPR induction dynamics following this stimulus, we depleted inositol in a yeast culture and assayed for Ire1 activity as reflected by the splicing of *HAC1* mRNA observed on Northern blots (Figure 1A, see Methods). After a lag phase—presumably the time required to exhaust residual inositol stores—*HAC1* mRNA splicing reached a maximal level by 120 min, and then declined during an adaptation phase to recover near basal levels by 240 min. Population growth slowed during the induction phase but was restored upon recovery (Figure S1A). Thus, the UPR indeed functions as a homeostat in response to inositol depletion: the lack of inositol triggers activation of the biosynthetic pathway via Ire1, which initially overshoots and then settles at a new basal level that meets the cells' needs to grow under the new conditions. In this example, our detection of *HAC1* mRNA splicing was not sensitive enough to detect a difference between the starting condition and the new basal level. However, blotting for the UPR target *INO1* mRNA, which encodes inositol 1-phosphate synthase required for *de novo* inositol synthesis, demonstrated that the readjusted level at the 240 min time point was elevated compared to the un-induced system (Figure 1A, right panel), as was the expression of a UPR reporter (Figure S1B).

To determine whether similar adaptation also occurs after Ire1 activation in response to other modes of UPR induction, we treated cells with DTT, a reducing agent that counteracts disulfide bond formation and thereby induces protein misfolding in the ER. Disulfide bonds are formed through a relay in which ER client proteins are initially oxidized by protein disulfide isomerase (PDI). PDI is in turn oxidized by the FAD-dependent oxidase Ero1, which is finally oxidized by molecular oxygen [28]. Both *PDI* and *ERO1* are UPR target genes, but since Ero1 directly passes the electrons to molecular oxygen, its abundance limits oxidative capacity. Thus, we reasoned that for moderate amounts of DTT, UPR-mediated induction of *ERO1* would compensate for the increased demand for oxidation, allowing Ire1 to deactivate.

To test this, we treated cells with a range of DTT concentrations. Cells treated with 5 mM DTT no longer proliferated, indicating the presence of a maximal ER stress beyond which cells can no longer compensate effectively even in the presence of a maximally active UPR (Figure 1B, black). By contrast, cells treated with 2.2 mM or 1.5 mM DTT continued to proliferate, albeit at rates decreased from control cells (Figure 1B, purple and green). To investigate whether these growth phenotypes correlated with the activation and deactivation of the UPR, we monitored Ire1 activation by measuring *HAC1* mRNA splicing as above (Figure S2). Consistent with the observed growth arrest, Ire1 activation was maximal and sustained in 5 mM DTT (Figure 1C, black): *HAC1* mRNA was spliced to its full extent 30 min after DTT addition and splicing was maintained at this high level for the duration of the experiment. By contrast, in cells treated with doses of 2.2 mM or 1.5 mM DTT, Ire1 deactivation occurred in 4 h and 2 h, respectively (Figure 1C, blue and green). Therefore, under non-saturating DTT conditions, cells show the same transient Ire1 activity that characterized the response to inositol depletion. Furthermore, the duration of that transient response increased along with the magnitude of the stress.

To ascertain that the Ire1 activation and deactivation phases are reflective of the regulation of UPR target genes, we measured the expression of a synthetic UPR-regulated GFP transcriptional reporter (TR) over time in cells treated with 1.5 or 2.2 mM DTT (Figure 1D, E, see Methods). In these cells, the TR was induced to dose-dependent plateaus after a lag of approximately 30 min. The lag is consistent with the time required for transcription, translation, and GFP chromophore maturation, while the plateaus reflect the accumulation of the long-lived GFP reporter protein (half-life >8 h). Induction of a natural UPR target promoter, *ERO1*, closely matched the response from the synthetic TR (Figure S3). Therefore, the expression of UPR target genes at any given time is reflected by the rate of GFP production, rather than its abundance. When plotted as a function of the rate of GFP production (dTR/dt; Figure 1E), the TR exhibited activation and deactivation phases at 1.5 and 2.2 mM DTT that mirrored the dynamics of upstream *HAC1* mRNA splicing (compare Figure 1C and 1E).

Taken together, the data shown in Figure 1 indicate that under different inducing stimuli, the UPR undergoes induction and adaptation phases that are reflected in the transient splicing activity of its sensor Ire1. Ire1 activity, in turn, is faithfully transmitted to the system's transcriptional output.

### Ire1^bipless^ Is Stress-Inducible But Can Organize in Small Foci in the Absence of Stress

**Figure 2:**
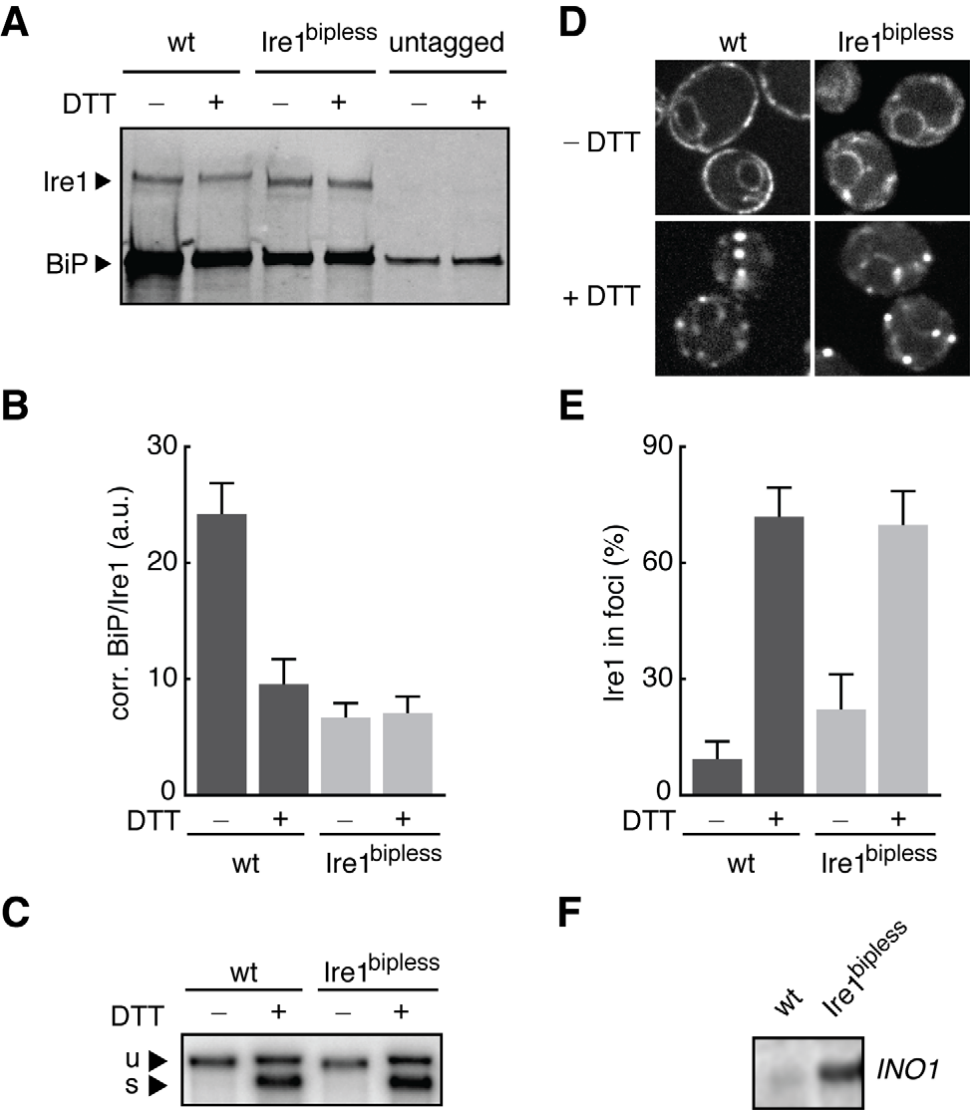
Ire1^bipless^ is stress-activated with no change to its association with BiP. (A) Ire1^bipless^ is a mutant of Ire1 lacking 51 amino acids containing the BiP interaction motif (Δ475–526). Cells bearing HA-tagged alleles of wild type Ire1 or Ire1^bipless^ were harvested before and after treatment with 5 mM DTT for 1 h. Cells were lysed and Ire1 and Ire1^bipless^ were immuno-precipitated with anti-HA agarose beads. The proteins eluted from the beads were resolved by SDS-PAGE, transferred to PVDF, co-incubated with anti-HA and anti-BiP antibodies followed by fluorophore-conjugated secondary antibodies, and scanned on the Li-Cor imager. BiP decreased its association with wild type Ire1 after treatment with DTT, while BiP did not change its association with Ire1^bipless^ after DTT treatment. Some BiP binds nonspecifically. (B) Three independent immunoprecipitation experiments were quantified after scanning with the Li-Cor. The ratio of BiP/Ire1, after subtraction of the nonspecific BiP signal as measured in the *Ire1*Δ cells, shows that BiP dissociates from wild type Ire1 in response to DTT, that Ire1^bipless^ binds to less BiP in the absence of stress than wild type Ire1 binds in the presence of DTT, and that Ire1^bipless^ does not change its association with BiP after treatment with DTT. (C) Cells bearing wild type Ire1 or Ire1^bipless^ were harvested before and after treatment with 5 mM DTT for 1 h, total RNA was purified, subjected to Northern blot analysis, and probed for *HAC1* mRNA. Wild type and Ire1^bipless^ displayed no differences in splicing: no *HAC1* mRNA was spliced in the absence of DTT and splicing was equally induced after treatment with DTT. (D) GFP-tagged alleles of wild type Ire1 and Ire1^bipless^ were expressed and imaged in the presence and absence of DTT. GFP domains are inserted between the transmembrane domain and the linker of the kinase domain on the cytoplasmic side of Ire1, as in [13]. Wild type Ire1 displays a diffuse perinuclear and cortical ER localization in the absence of stress and forms bright clusters after treatment of 5 mM DTT for 1 h. Ire1^bipless^ displays similar perinuclear and cortical localization in the absence of stress, but with small clusters in some cells. After DTT treatment, Ire1^bipless^ forms clusters like the wild type. (E) Quantification of Ire1 clustering shows that Ire1^bipless^ forms more foci in the absence of stress than wild type, but forms clusters equal to the wild type after treatment with 5 mM DTT for 1 h. (F) Wild type and ire^bipless^ cells in the absence of stress probed for basal expression of *INO1* mRNA expression.

To assess whether the activation and adaptation properties of Ire1 are dependent on BiP binding and dissociation, we expressed a mutant form of Ire1, Ire1^bipless^, lacking a 51 amino acid segment (Ire1^Δ475–526,GKSG^) that contains the BiP binding site (see Methods, Tables 1, 2). While similar to the Ire1^ΔV^ mutant described in [22], Ire1^bipless^ retains 10 amino acids defined in the crystal structure of the core lumenal domain [24] that were deleted in Ire1^ΔV^. As previously reported, wild type Ire1 associated with BiP in a co-immunoprecipitation assay in the absence of ER stress (Figure 2A, B) but the association diminished when cells were treated for 1 h with 5 mM DTT (Figure 2A, B). By contrast, no change in the association of Ire1^bipless^ and BiP was observed between stressed and unstressed cells (Figure 2A, B). The residual binding of BiP to Ire1^bipless^ is likely due to non-specific absorption of the notoriously sticky chaperone (Figure 2A, B). As the amount does not change between UPR-induced and uninduced cells, this residual interaction does not reflect a physiologically important regulatory interaction.

**Table 1:** Plasmids used in this study.

Plasmid	Description	Marker
pDEP005	SR, pRS305-Phac1-h5′-GFP-h3′	LEU2
pDEP007	Ire1-GFP, wt IRE1-GFP in pRS305	LEU2
pDEP010	Ire1-mCherry, wt IRE1 in pRS306	URA3
pDEP017	TR, pRS304-4×UPRE-GFP	TRP1
pDEP044	2 µ plasmid, wt IRE1 in pRS423	HIS3
pDEP045	2 µ plasmid, Ire1bipless in pRS423	HIS3
pDEP049	Ire1^bipless^, pRS306-PIre1-Ire1^bipless^	URA3
pDEP053	Ire1^bipless^-GFP, pRS306-PIre1-Ire1^bipless^-GFP	URA3
pDEP060	Ire1^biipless^-mCherrry, pRS305-Ire1bipless-mCherry	LEU2

**Table 2:** Yeast strains used in this study.

Yeast Strain	Description	Markers Used
YDP001	wild type, CRY1, w303a derivative	none
YDP002	ΔIre1, CRY1 ΔIre1::KAN	KANr
YDP003	wt SR, CRY1 SR::LEU	LEU
YDP005	wt TR, CRY1 TR::TRP	TRP
YDP007	Ire1-GFP, ΔIre1, Ire1-GFP::LEU	LEU
YDP010	Ire1-mCherry, ΔIre1, Ire1-mCherry::URA	URA
YDP012	FRET, ΔIre1, Ire1-GFP::LEU, Ire1-mCherry::URA	LEU, URA
YDP015	Δhac1, Δhac1::TRP	TRP
YDP016	Δhac SR, Δhac1::TRP, SR::LEU	LEU, TRP
YDP020	Ire1^bipless^, ΔIre1::KAN, Ire1^bipless^::URA	KANr, URA
YDP021	Ire1^bipless^ SR, ΔIre1::KAN, Ire1^bipless^::URA, SR::LEU	KANr, URA, LEU
YDP025	Ire1^bipless^-GFP, ΔIre1::KAN, Ire1^bipless^-GFP::URA	KANr, URA
YDP030	Ire1^bipless^-mCherry ΔIre1::KAN, Ire1^bipless^-mCherry::LEU	KANr, LEU
YDP036	Ire1^bipless^ FRET, ΔIre1::KAN, Ire1^bipless^-GFP::URA,	KANr, URA, LEU
	Ire1bipless-mCherry::LEU	

To determine whether the diminished association between Ire1^bipless^ and BiP impacts Ire1 activation, we measured *HAC1* mRNA splicing in wild type cells and cells expressing Ire1^bipless^ grown in the presence and absence of 5mM DTT for 1 h (Figure 2C). In both wild type and Ire1^bipless^ cells, no detectable *HAC1* mRNA was spliced in the absence of stress, and splicing was identically induced in the two strains after treatment with DTT. These data refute any model that poses modulation of the BiP•Ire1 association as the exclusive regulator of Ire1 activity.

Next, we investigated the subcellular localization of Ire1^bipless^ in the presence and absence of ER stress. In response to ER stress, wild type Ire1 oligomerizes in clusters in the ER membrane that appear as discrete foci in fluorescence microscopy images [14],[15]. Similar to wild type GFP-tagged Ire1, GFP-tagged Ire1^bipless^ displayed cortical and perinuclear ER localization in the absence of stress and formed bright foci in cells treated for 1 h with 5 mM DTT (Figure 2D). Quantification revealed that Ire1^bipless^ formed foci of equal magnitude to the wild type protein upon UPR induction. In unstressed cells, however, Ire1^bipless^ displayed a 2-fold increase in the level of clustering compared to wild type Ire1 (Figure 2E), and the foci exhibited considerable cell-to-cell variability (Figure S4, see Discussion).

The increased clustering of Ire1^bipless^ did not apparently lead to activation, since a Northern blot of total RNA from cells bearing Ire1^bipless^ did not show detectable amounts of spliced *HAC1* mRNA in the absence of stress (Figure 2C). We considered it possible that splicing occurred at a level below the detection limit of the Northern blot assay. This reasoning is supported by Northern blots for *INO1* mRNA, which is a more sensitive indicator of UPR induction as demonstrated above (Figure 1A, right). Indeed, *INO1* mRNA was significantly elevated in cells expressing Ire1^bipless^ as compared to cells expressing wild type Ire1 under non-inducing conditions (Figure 2F). Furthermore, there is a notable increase in the basal signal from a UPR reporter in unstressed Ire1^bipless^ cells (Figure S5). Thus, UPR signaling in Ire1^bipless^ cells is leaky.

### Ire1^bipless^ Cells Are Sensitized to Low Levels of ER Stress

The propensity of Ire1^bipless^ to form small clusters in the absence of stress prompted us to ask if cells bearing Ire1^bipless^ would be more sensitive than wild type to low levels of stress. To test this notion, we expressed a GFP splicing reporter (SR), in which the first exon of the *HAC1* open reading frame is replaced by GFP (Figure S6A). The *HAC1* intron represses translation of the mRNA, so GFP is only produced once active Ire1 removes the intron. Using flow cytometry, the SR allowed us to precisely quantify Ire1 activity over time in wild type and Ire1^bipless^ cells. The SR did not compete with endogenous *HAC1* mRNA for Ire1 when wild type cells were treated with 5 mM DTT for 1 h (Figure S6B), and similar to the TR, the GFP encoded by the SR decayed with a half-life of >8 h.

**Figure 3:**
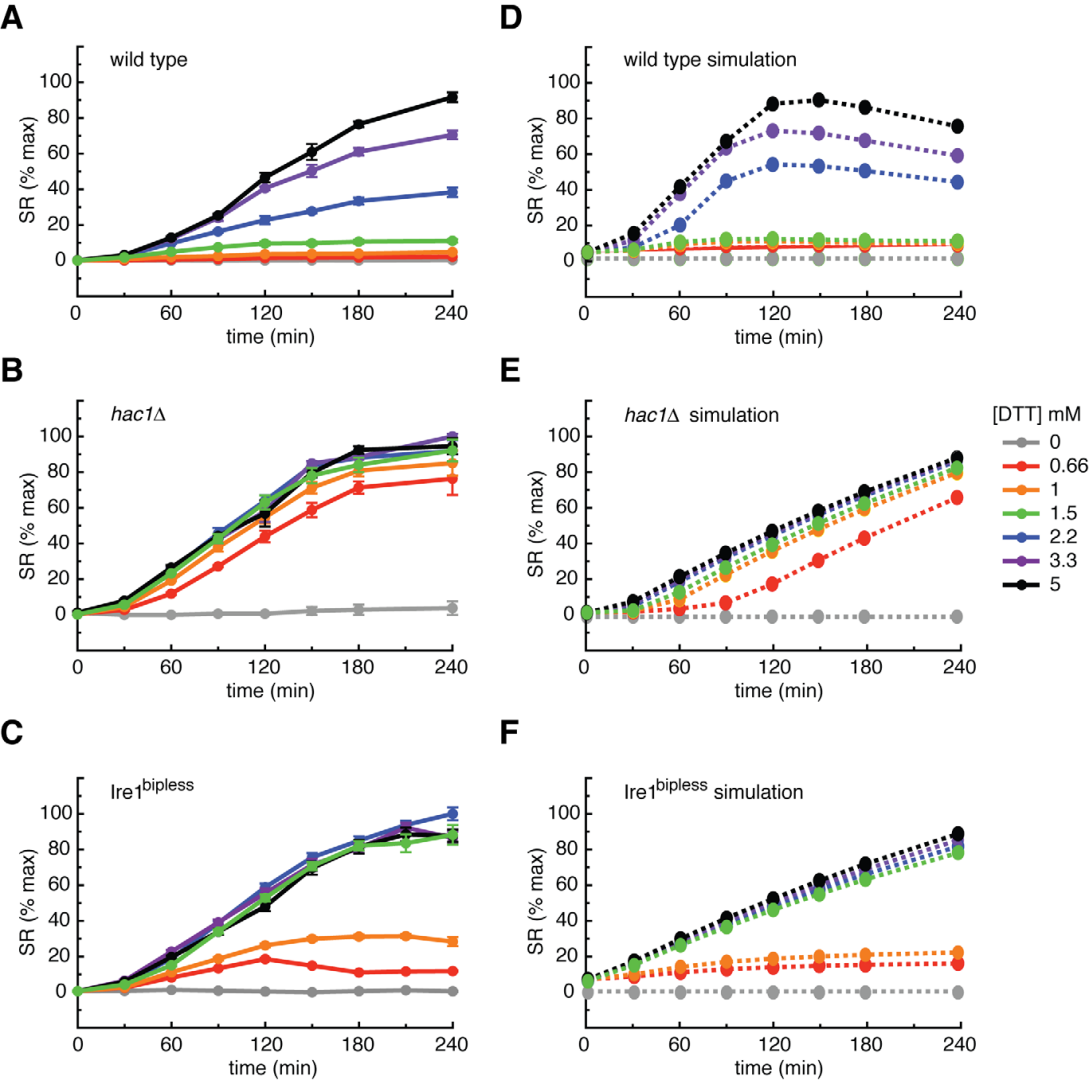
Experimental and simulated DTT titration time courses in wild type, *hac1*Δ, and Ire1^bipless^ cells. (A) Wild type cells expressing the GFP splicing reporter (SR) were treated with doses of DTT spanning the active concentration range, sampled over time, and their fluorescence was measured by flow cytometry. The SR, like the TR, reached dose-dependent plateaus due to the >8 h half life of GFP. (B) *hac1*Δ cells expressing the SR were treated as above. *hac1*Δ cells were hypersensitive to DTT and saturate the reporter at all experimental doses. (C) Ire1^bipless^ cells expressing the SR were treated as above and showed increased sensitivity to DTT compared to the wild type, responding to 0.66 mM DTT and saturating at 1.5 mM DTT. (D) Simulations of the “wild type” model. The architecture of the model, described in the text and depicted in Figure 4A, includes BiP binding to Ire1 and negative feedback. When the model includes a cooperative Ire1 deactivation term (described in text), it recapitulated the wild type DTT titration time course. (E) Simulations of the “*hac1*Δ” in which the negative feedback terms have been removed captured the hypersensitivity observed experimentally. (F) Simulations of the “Ire1^bipless^” model in which the Ire1/BiP interaction terms have been removed revealed the increased DTT sensitivity compared to the wild type.

When wild type cells expressing the SR were treated with increasing concentrations of DTT, the SR was induced to dose-dependent plateaus (Figure 3A), and the rate of GFP production displayed the peak and decline behavior characteristic of the splicing of endogenous *HAC1* mRNA (dSR/dt; Figure S7A). Consistent with the data shown in Figure 1, cells expressing wild type Ire1 were insensitive to DTT at concentrations below 1.5 mM as apparent from the absence of SR induction. By contrast, *hac1*Δ cells were hypersensitive to DTT: they induced the SR to near maximal levels at all doses (Figure 3B), and the rate of GFP production remained high until the reporter saturated (Figure S7B). In the absence of *HAC1*, Ire1 activation fails to initiate a transcriptional response, and the stress is never alleviated.

Interestingly, Ire1^bipless^ cells showed an intermediate SR phenotype. Ire1^bipless^ cells were more sensitive to DTT than wild type cells, becoming activated at 0.66 mM DTT and saturated at 1.5 mM DTT (Figures 3C, S7C). These data are consistent with the notion that increased clustering in Ire1^bipless^ cells in the absence of DTT is coupled with sensitization, which allows activation at low levels of stress.

### A Computational Model of Ire1 Regulation Recapitulates the Enhanced Sensitivity of the UPR in Ire1^bipless^ Cells

**Figure 4:**
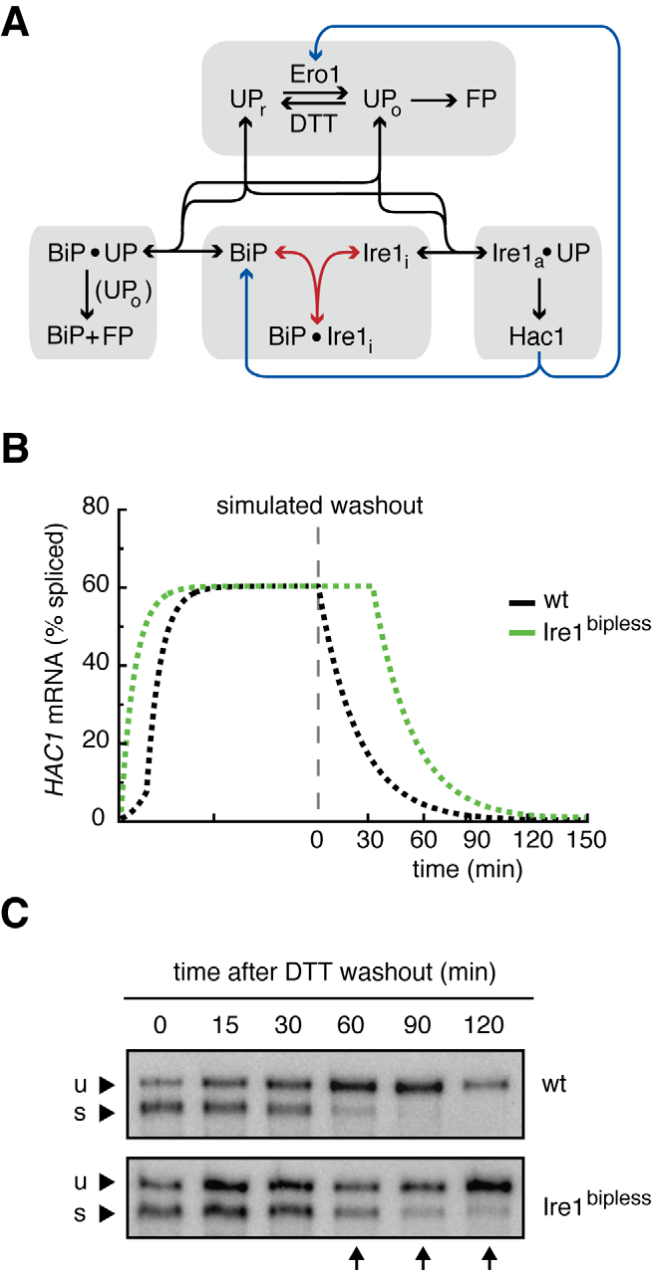
Model architecture, prediction and experimental validation. (A) The molecular interactions that comprise the model. See the supplement for complete modeling details. Ire1 can exist in three states: (1) inactive monomer (Ire1_i_, middle lower box), (2) inactive in complex with BiP (Ire1_i_•BiP, middle lower box), and (3) active in complex with an unfolded protein (Ire1_a_•UP, lower right box). Either reduced (UP_r_) or oxidized (UP_o_) can bind to and activate Ire1, but UP_o_s quickly become folded proteins (FP, upper box and lower left box). The amount of UP_r_s and UP_o_s is determined by the flux of unfolded proteins and the red/ox potential, defined here as the ratio of Ero1/DTT. Active Ire1 in complex with unfolded proteins produces the Hac1 transcription factor, which induces the production of Ero1 and BiP. BiP can also exist in three states: (1) monomer (BiP, middle lower box), (2) bound to Ire1_i_ (BiP•Ire1_i_), and (3) in complex with unfolded proteins (BiP•UP). BiP can bind to both UP_r_ and UP_o_, but only aids in the folding of UP_o_ (bottom left box). The blue arrows indicate the feedback terms that are removed in the “*hac1*Δ” model, and the red arrows indicate the Ire1/BiP interaction terms that are removed in the “Ire1^bipless^” model. (B) Simulations “wild type” and “Ire1^bipless^” cells treated with 5 mM DTT for 100 min and then the DTT is suddenly removed predict a deactivation delay for Ire1^bipless^ cells: “wild type” cells immediately began to deactivate while Ire1^bipless^ continued activity for ∼30 min after DTT withdrawal. (C) Wild type and Ire1^bipless^ were treated with 5 mM DTT for 1 h, filtered, washed, and resuspended in fresh media lacking DTT and sampled over time. Samples were assayed for *HAC1* mRNA splicing by Northern blot to measure Ire1 activity. Consistent with the simulations, wild type cells deactivated after 90 min while Ire1^bipless^ cells deactivated after 180 min.

To validate that our data are consistent with a model of Ire1 regulation that includes interactions with unfolded proteins and BiP and to provide hypotheses for how BiP could specifically contribute to Ire1 regulation, we built a computational model of the UPR with the following assumptions (see Text S1). Ire1 can exist in one of three states: (i) as a free inactive monomer, (ii) as an inactive complex bound to BiP, or (iii) as an active complex bound to an unfolded protein (Figure 4A). Further, free BiP can bind to unfolded proteins and either productively aid in their folding or nonproductively dissociate. Unfolded proteins are either reduced or oxidized depending on the redox potential of the ER and must be oxidized in order to fold. In the model, the redox potential is set by the ratio of DTT to Ero1. When bound to an unfolded protein, the active Ire1 complex initiates the production of the Hac1 transcription factor, which in turn increases the production of BiP and Ero1 to close the UPR feedback loop. To explicitly model the measured experimental output (GFP fluorescence), the active Ire1 complex was set to trigger the production of a simulated SR in addition to producing Hac1. We extracted available model parameters from the literature and fitted remaining parameters to a subset of the experimental data (Figure S8, see Supporting Information for details). Using this “wild type” model as a baseline for comparison, we generated a “*hac1*Δ” model in which no induced production of BiP or Ero1 exists and an “Ire1^bipless^” model in which the interaction between Ire1 and BiP is disabled (Figure 4A).

The functional form of the dissociation of the active Ire1/unfolded protein complex was a modeling choice. Significantly, a model in which this dissociation was assumed to be linear did not reproduce the difference between the wild type and Ire1^bipless^ when the SR time courses were simulated (Figure S9). Instead, a nonlinear, cooperative dissociation function of the active Ire1-unfolded protein complex was required to recapitulate the data; i.e., the dissociation rate of the active Ire1-unfolded protein complex must decrease in proportion to the concentration of the active oligomeric complex raised to a power greater than one. Given that Ire1 signals by clustering into foci, this nonlinear dissociation function can be thought of as a consequence of having to disassemble a cooperative enzyme complex (Figure S10, see Discussion). When simulated with such nonlinear dissociation of the active Ire1 complex, the model robustly recapitulated the DTT titration time course results in wild type, *hac1*Δ, and Ire1^bipless^ cells (Figure 3D–F). When the SR time course was simulated with the wild type Ire1 model, doses of DTT of 1.5 mM and below produced less than 10% activity, 2.2 mM DTT produced an approximately half-maximal response, 3.3 mM DTT produced a response of approximately 75% of the maximum, and 5 mM DTT produced a near saturating response (Figure 3D). By contrast, simulation of the *hac1*Δ model produced near saturating responses to all doses, recapitulating the hypersensitivity measured in vivo (Figure 3E). Furthermore, simulation of the Ire1^bipless^ model yielded an intermediate phenotype in which 0.66 mM DTT produced 15% activity, and doses of 1.5 mM DTT and above saturated the response (Figure 3F). Importantly, this agreement between the model simulations and experimental data was an emergent property of the functional interactions in the system, which arose independently of the choice of parameter values (Figures S11, S12).

### In Silico Modeling Predicts a Role for BiP in Ire1 Deactivation Kinetics

In addition to accounting for the increased sensitivity of Ire1^bipless^ compared to the wild type in the DTT titration time course experiments, our computational model predicted that Ire1^bipless^ should exhibit delayed shutoff dynamics compared to the wild type after DTT is removed (Figure 4B).

This prediction can be rationalized in intuitive terms. When DTT is removed, disulfide bonds can form and proteins can mature. Thus the concentration of the ligand for Ire1 activation starts to decrease, and individual Ire1 molecules dissociate from the active oligomer. When wild type Ire1 dissociates, it can either rejoin the signaling complex (through interaction with an unfolded protein), or it can bind to BiP. Therefore, Ire1 deactivation proceeds rapidly since the inactive free form can be sequestered away by binding to BiP. In contrast, Ire1^bipless^ lacks the ability to interact with BiP. Thus, while DTT removal will still prompt the dissociation of Ire1 from the active oligomer as the concentration of unfolded proteins decreases, the inability of Ire1^bipless^ to bind to BiP increases the probability that an inactive Ire1^bipless^ monomer will be recaptured by an unfolded protein and reactivate. As a result, Ire1^bipless^ deactivation would proceed more slowly than that of wild type Ire1.

To test this prediction experimentally, we performed a DTT washout experiment in which wild type and Ire1^bipless^ cells were treated with 5 mM DTT for 1 h to fully activate Ire1 in both strains. Subsequently, DTT was removed by filtration, cells were washed and resuspended in fresh media, and samples were collected over time to assay for *HAC1* mRNA splicing by Northern blot (Figure 4C). Additional samples of wild type cells were collected to assay for the association of Ire1 and BiP by immunoprecipitation (Figure S13). Confirming the model predictions, we found that while Ire1 deactivated after 60 min in the wild type, Ire1^bipless^ retained activity for 120 min. As expected, Ire1 deactivation correlated with re-association with BiP (Figure S13). These results point to a role for BiP binding in promoting Ire1 deactivation once stress has been alleviated.

### FRET Measurements of Ire1 Oligomers Reveal a Mechanistic Role for BiP in Ire1 Deactivation

To pursue the mechanism through which Ire1 deactivation proceeds, we hypothesized that, since Ire1 signals through assemblies of high-order oligomers, BiP binding may sequester breakaway Ire1 monomers, therefore promoting de-oligomerization of active Ire1 complexes. If this were the case, Ire1^bipless^ cells should exhibit slower disappearance of Ire1 oligomers than wild type cells upon removal of stress.

**Figure 5:**
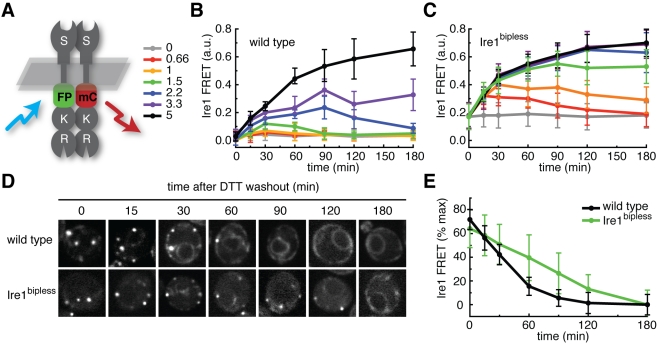
FRET measurements of wild type Ire1 and Ire1^bipless^. (A) Cartoon of Ire1 FRET. GFP- and mCherry-tagged versions of Ire1 or Ire1^bipless^ were co-expressed and cells were imaged by confocal microscopy. GFP and mCherry domains are inserted between the transmembrane domain and the kinase linker on the cytoplasmic side of Ire1, as in [13]. When exposed to blue light (488 nm) the GFP is excited, and if it is within a few nm of mCherry, it can excite mCherry instead of emitting green light. This transferred energy is emitted by mCherry as red light and can be measured as a FRET signal. (B) DTT titration time course measured by FRET in wild type cells. Ire1 displayed transient oligomerization after treatment with 2.2 mM or 1.5 mM DTT, and sustained oligomerization in response to 5 mM DTT. Doses are indicated in (C). (C) DTT titration time course measured by FRET in Ire1^bipless^ cells. Ire1^bipless^ displayed sustained oligomerization after treatment with 2.2 mM or 1.5 mM DTT, and transient activation after treatment with 0.66 and 0.99 mM DTT. (D) Cells expressing FRET pairs of wild type Ire1 (top panels) or Ire1^bipless^ (bottom panels) were treated with 5 mM DTT for 1 h and subsequently washed, resuspended in fresh media, and imaged by confocal microscopy. (E) Quantification of FRET signal from DTT washout experiment. Wild type Ire1 de-oligomerized completely by 90 min, while Ire1^bipless^ did not fully de-oligomerize for 180 min.

To directly test this hypothesis, we co-expressed GFP- and mCherry-tagged versions of Ire1 or Ire1^bipless^ and employed a microscopy-based fluorescence resonance energy transfer (FRET) assay [29] to quantify Ire1 self-association (Figures 5A, S14, see Methods). In an otherwise wild type scenario, the FRET signal displayed a broad dynamic range, from 0.01 a.u. (s.e.m. = 0.02, *n* = 36) in untreated cells in which the Ire1 fluorescence displayed a diffuse ER localization to 0.73 a.u. (s.e.m. = 0.06, *n* = 41) in cells treated with 5 mM DTT for 4 h, in which Ire1 is maximally clustered into foci (Figure S6B). In Ire1^bipless^ cells, the basal FRET signal in the absence of DTT was elevated to 0.17 a.u. (s.e.m. = 0.09, *n* = 53), but the maximum FRET signal in the presence of DTT (0.71 a.u., s.e.m. = 0.08, *n* = 32) was comparable to wild type. As expected, wild type cells displayed transient increases in FRET signal that returned to baseline levels over the course of the experiment after treatment with 2.2 or 1.5 mM DTT (Figure 5B, C). In contrast, Ire1^bipless^ cells were sensitized and displayed transient increases in FRET signal only when treated with 0.66 mM or 0.99 mM DTT but showed persistent strong FRET signal when treated with 1.5 mM or 2.2 mM DTT. These data recapitulate the role of BiP in buffering the Ire1 to low levels of stress (Figure 3).

To assess the role of BiP in the de-oligomerization of Ire1, we performed a DTT washout experiment and measured Ire1 FRET over time in wild type and Ire1^bipless^ cells (Figure 5D, E). After treatment of both strains with 5 mM DTT for 1 h, we washed the cells in fresh media lacking DTT and imaged the cells over time. Consistent with the deactivation kinetics of wild type and Ire1^bipless^ cells as measured by Northern blot, wild type Ire1 de-oligomerization proceeded rapidly and the FRET signal returned to baseline after 60 min. By contrast, the Ire1^bipless^ FRET signal remained higher than basal levels at 120 min. Taken together, these data indicate that BiP binding to Ire1 contributes to the efficient de-oligomerization of active Ire1 complexes.

## Discussion

In this work, we investigated the homeostatic properties of the UPR in response to a range of physiological stress levels. Using time-resolved measurements of the induction and adaptation kinetics of the wild type UPR and a mutant UPR in which the sensor molecule Ire1 is not modulated by the chaperone BiP, we established a model for dynamic UPR regulation. In this model, Ire1 is principally activated when unfolded proteins bind to it directly. In a dynamic equilibrium, binding to unfolded proteins pulls Ire1 into oligomeric clusters and away from the chaperone BiP. Oligomerization, which occurs as a direct consequence of unfolded protein binding to Ire1's lumenal domain, is necessary and sufficient for Ire1 activation, and as such is the central control point in the UPR. Rather than regulating the first step of Ire1 activation, BiP provides superimposed modulation of the UPR's dynamic properties. Specifically, BiP assumes a dual role in which it simultaneously acts as a buffer to reduce the system's sensitivity to low stress levels and as a timer to tune the response time to the magnitude of stress by assisting in Ire1 deactivation once homeostasis is restored to the ER. The model establishes the UPR as a dynamic system whose capacity is adjusted to efficiently counteract a large spectrum of stress magnitudes and suggests a long-sought role for BiP binding to Ire1.

### The UPR Is a Homeostat

When cells experience protein folding stress in the ER, the UPR is activated to increase the ER's folding capacity. For manageable stress magnitudes, the UPR is capable of restoring folding homeostasis. However, if the magnitude of the stress surpasses the capacity of the UPR, yeast cells sustain maximal Ire1 signaling and cease to proliferate (Figure 1B, C). Within the physiological regime of ER stress, the response of Ire1 to moderate DTT inputs (1.5 mM and 2.2 mM DTT, Figure 1C) displayed transient activation dynamics, followed by adaptation to near basal levels. Interestingly, the duration of Ire1 activity—not the maximal amplitude of its activity—correlated with the magnitude of the stress. Since the Hac1 transcription factor is short-lived, the length of the Ire1 activation pulse should determine the duration of UPR target gene activation by Hac1 [18],[19]. This in turn determines the volume of the ER and the concentration of ER chaperones, components of the degradation machinery, and other cytoprotective proteins that are produced to combat the stress. This mode of signal regulation in which the duration of the output matches the magnitude of the input is known in engineering as “pulse-width modulation.” It is widely employed to reduce noise in engineered control systems by transforming an analog signal (amplitude) into a digital all-or-none pulse of varying length [30].

Although in principle real-time information about the folding status of the cell could be conveyed exclusively through the interaction of unfolded proteins with Ire1 to determine the duration of UPR induction, we find that BiP plays an important role in modulating the length of the Ire1 activation pulse (Figures S6A,C, 5B,C). Perhaps this modulating role of BiP reflects the necessity for precise tuning of the Ire1 pulse beyond what can be achieved through Ire1 and unfolded proteins alone. Interestingly, it was recently shown that a mutant of mammalian Ire1α shares salient properties with Ire1^bipless^: it does not bind to BiP, retains ER stress inducibility, and displays increased basal activity [31]. Therefore, it seems likely that the role of BiP in buffering Ire1 oligomerization is conserved in mammalian cells. Moreover, as the transmembrane kinase PERK, which in metazoan cells functions in a parallel UPR signaling branch to Ire1, shares close sequence homology to Ire1's lumenal domain, lessons learned for Ire1 modulation by BiP are likely to also apply to PERK regulation.

Precise tuning, and subsequently the buffering role of BiP, becomes all the more important since the UPR is linked to crucial cell fate decisions such as commitment to apoptosis [32]. The decision to commit to apoptosis might depend directly on the time of exposure to stress or on a thresholding mechanism through which either the extent of cellular damage or UPR machinery are assessed. Both scenarios would translate into an enhanced commitment to apoptosis in the absence of BiP modulation of Ire1.

### BiP Buffers Ire1's Switch-Like Activity

As detailed above, precision homeostasis in the UPR requires the pathway-specific interaction of Ire1 and BiP. Disruption of this interaction in vivo leads to increased sensitivity to low levels of stress (“leakiness”), coupled to slower deactivation of Ire1 once stress is removed (Figure 4C). By using FRET to measure Ire1 self-association, we found that BiP performs these functions by aiding Ire1 de-oligomerization (Figure 5C–E). In vitro, Ire1 functions as a cooperative enzyme with a Hill coefficient >8, and the active species are large oligomers [12]. This high cooperativity could translate in vivo to a switch-like response of Ire1 to small changes in the concentration of unfolded proteins. For example, it follows from basic principles of enzyme kinetics that if Ire1 signals in clusters of 16 molecules, a mere 35% increase in unfolded proteins would cause Ire1 to go from 10% to 90% active. In this light, BiP's role as a binding partner that desensitizes Ire1 can be viewed as a gatekeeper that prevents triggering of the Ire1 activation switch following small or transient fluctuations in the local concentration of unfolded proteins. By doing so, BiP works to ensure that Ire1 is only activated when the stress is sufficient to warrant a response, thus improving information quality in the signaling pathway [33].

It is formally possible that in addition to loss of its UPR-specific BiP interaction Ire1^bipless^ retains its ER-stress dependent activation, yet displays altered activation dynamics due to non-native conformational interactions. However, since Ire1^bipless^ oligomerizes and activates in a ligand-specific manner to the same extent as wild type Ire1, we contend that in the simplest scenario, Ire1^bipless^, like the previous “bipless” mutant Ire1^ΔV^ [22],[25], is a structurally sound molecule that is activated by the same mechanism that activates wild type Ire1.

Though similar to Ire1^bipless^, Ire1^ΔV^ was not shown to be hypersensitive to DTT or to deactivate after washout with delayed kinetics [22]. However, Ire1^ΔV^ did display hypersensitivity to heat shock and delayed deactivation kinetics in response to ethanol [22]. While the discrepancies between Ire1^bipless^ and Ire1^ΔV^ may be due to differences in experimental resolution, the elevated response of Ire1^ΔV^ to heat shock and ethanol is consistent with the notion that BiP buffers Ire1 to these mild ER stresses.

### Ire1 Regulation Reconstituted in Silico Holds Clues to the Mechanisms of Ire1 Modulation by BiP

Our study of the intricate UPR dynamics was guided by a computational model which was able to recapitulate our data and generate useful predictions. In the model, BiP serves as a buffer to the pool of inactive Ire1. By binding to free Ire1, BiP sequesters the inactive form of Ire1 and both prevents activation at low levels of stress and promotes deactivation once the stress has been overcome (Figures 3D–F, 4B).

This mechanism of Ire1 activation in our model contrasts with the two-step Ire1 activation model [15], in which unfolded proteins first trigger BiP dissociation from Ire1 to induce oligomerization, and subsequently bind to the oligomers to activate signaling. As opposed to separating oligomerization and activation into two steps, our model treats unfolded protein binding as the single activating step; Ire1 is in dynamic equilibrium with BiP and unfolded proteins, and its unfolded protein bound state is active. Thus, BiP dissociation, rather than triggering oligomerization, yields monomeric Ire1, which can then either bind to an unfolded protein and activate or re-bind to BiP. We note that the small Ire1^bipless^ foci that formed in the absence of stress resulted in increased expression of *INO1* mRNA and increased basal levels of UPR reporter fluorescence (Figures 1A, S5). Thus, we never observed inactive foci, in support of our model that oligomerization and activation occur in the same step.

In addition to this different mechanism of Ire1 activation, our model also proposes a mechanism for Ire1 deactivation. Since BiP and unfolded proteins compete for Ire1, BiP serves as a buffer that allows rapid deactivation of Ire1 as the concentration of unfolded proteins decreases. Finally, in contrast to the static picture of Ire1 activation presented in the two-step model, we present a time-resolved, quantitative model that accurately portrays Ire1 activation in response to any dose of DTT over time in its activation and adaptation phases.

While the computational model reflects our current understanding of Ire1 regulation, it is likely to be an oversimplification. Next generation models could easily improve the verisimilitude by including additional ER processes that are not currently represented in the model (such as glycosylation, ERAD, and BiP's ATP hydrolysis cycle) or better constraining the model parameters by targeted measurements. Yet even with increasing mechanistic detail the requirement for cooperative Ire1 deactivation is likely to persist (Figure S9). This feature, modeled as decreasing Hill function of active Ire1 molecules, is consistent with the notion that Ire1 signals through assemblies of high-order oligomers. As Ire1 oligomers grow in size or number, the percentage of Ire1 molecules that have the ability to be deactivated decreases as many molecules become captured inside macromolecular assemblies. Such cooperativity in Ire1 deactivation can be depicted intuitively as a simple steric consequence of Ire1 oligomerization (Figure S10).

Interestingly, this cooperativity can also be invoked to interpret the increased variability in foci formation in the Ire1^bipless^ mutant cells (Figures S4 and S15). BiP's role can be thought of as a vehicle to help Ire1 traverse the threshold-like inactivation curve. In a wild type cell where focus formation might initiate stochastically, the presence of BiP can accelerate the dissociation of the foci. However, in an Ire1^bipless^ mutant, any stochastically formed focus would be stable for a longer time (Figure 5C–E). If focus dissolution is an all-or-none process, an extreme scenario is one where Ire1 focus formation in wild type and Ire1^bipless^ cells occurs as a pulse train whose low frequency of activation is the same in both populations. However, the duration of each pulse would be longer in Ire1^bipless^ than in wild type cells. This simplified scenario would result in modest differences in foci formation as averaged over the population since the activation probability is itself low. It would nonetheless result in large variations around this average exhibited by individual cells. According to this view, BiP buffering would ensure that activated Ire1 signaling centers assume a more homogeneous size, providing for a consistent input/output relationship and consistent deactivation kinetics. As such, BiP buffering fine-tunes the UPR by filtering noise from the signal transmission process, thereby increasing the information content of the signal and improving the cell's homeostatic control of the ER.

This mode of regulation by which a free pool of a protein is buffered by chaperones may be a widely used mechanism in biology. For example, many kinases interact with cytosolic chaperones, and kinase signaling receptors that oligomerize during activation may hence be buffered similarly. Moreover, dynamic protein assemblies, such as clathrin coats or SNARE complexes, utilize chaperone interactions to aid disassembly [34],[35]. Insights gained from our understanding of the functional consequences of the interaction between BiP and Ire1 may therefore be generally applicable to many other systems, in which protein oligomers have to form and be broken down again in a highly controlled manner.

## Methods

### Strains and Cell Growth

Reporter constructs and mutant alleles are genomically integrated into wild type or mutant strains. All experiments were conducted in complete, synthetic media (2×SDC: yeast nitrogen base, glucose, complete amino acids).

### Reporter Constructs

#### TR (transcriptional reporter)

The TR is GFP under the control of a crippled *cyc1* promoter, containing 4 repeats of a UPR-responsive cis element (4×UPRE).

#### SR (splicing reporter)

The SR is a reporter of Ire1 endonuclease activity. It is expressed from the native *HAC1* promoter and identical to the *HAC1* mRNA except that the first exon has been replaced by that of GFP. The intron, splice sites, and untranslated regions are identical to the *HAC1* mRNA.

#### Ire1 imaging and FRET reporters

All fluorophore-tagged versions of Ire1 and Ire1^bipless^ have the fluorescent protein (GFP or mCherry) inserted between the transmembrane domain and the cytoplasmic linker that connects the kinase domain to the transmembrane domain, as in [13].

#### HA-Tagged Constructs

Ire1 and Ire1^bipless^ were c-terminally HA-tagged for immunoprecipitation and immuno-detection.

### Construction of Ire1^bipless^ and Expression in Yeast Cells

Ire1^bipless^ is an allele of Ire1 that lacks the 51 amino acid juxtamembrane segment of the lumenal domain. This region is not in the crystal structure of the lumenal domain (Credle et al. [24]). Amino acids 475–526 of Ire1 were removed by 2-step PCR cloning and replaced with a 4 amino acid linker (Gly-Lys-Ser-Gly) on an episomal yeast plasmid (pRS315). The resulting positive, sequenced clone (*Ire1^bipless^*) was sub-cloned onto integrative plasmids (pRS305, pRS306), transformed into *Ire1*Δ cells (YDP002), and shown to complement for growth in the absence of inositol. Imaging constructs of Ire1^bipless^ (GFP- and mCherry-tagged) were created by sub-cloning from the sequenced plasmid into the integrative wild type Ire1-GFP and Ire1-mCherry plasmids used for the FRET experiments. All experiments except the immunoprecipitations were conducted with genomically integrated Ire1^bipless^ constructs.

### Northern Blot Analysis

We cultured cells in 2×SDC media to OD_600_ = 0.4, collected 50 ml per sample, washed cells in 1 ml 2×SDC and stored pellets at −80°C. Total RNA was extracted by resuspending cells in AE buffer (50 mM NaOAc, pH 5.2, 10 mM EDTA in DEPC-treated water), adding SDS to 1% and acid phenol (pH ∼4) (Fisher) to 50%, and heating at 65°C for 10 min. After spinning out the cell remains, we added chloroform and separated by centrifuging in phase-lock tubes (5 Prime). We precipitated the RNA with ethanol, washed with ethanol, and finally dissolved in 50 µl DEPC water.

RNA samples were quantified by spectrophotometry, added to loading buffer (1×E/formamide/formaldehyde/ethidium bromide/bromphenol blue), and heated at 55°C for 15 min. Samples were cooled on ice for 5 min and loaded. The gel is 1.5% agarose/20% formaldehyde/1×E and is run for 270 min at 100 V. Gels were transferred to nitrocellulose by wicking in 10× SSC for 24 h, and RNA crosslinked with 150 J. Blots were pre-hybridized in Church buffer for 3 h at 65°C, and hybridized overnight with random primer-generated probes from a *HAC1* PCR product that incorporated α-^32^P-CTP using GE ready-to-go beads. Blots were washed in 2× SSC, sealed in plastic, exposed to phosphor-imager screens overnight, imaged with the storm scanner, and quantified with ImageQuant software.

### Titration Time Courses and Flow Cytometry

We cultured cells bearing the SR or TR at 30°C in 2× SDC in 96 well deep well plates in an Innova plate shaker at 900 rpm. DTT stocks were made fresh from powder stored at 4°C for each experiment, and always 1 M in 10 ml. From this stock kept on ice, we prepared fresh 5× working stocks to start the experiment by diluting DTT in 1 step into 2× SDC to 37.5 mM (5×7.5 mM) in 10 ml. This 37.5 mM working stock was serially diluted by 1.5-fold increments (6 ml + 3 ml SDC) 10 times to span the range 0.13–7.5 mM. Every 2 h throughout the experiment, we repeated the full dilution series from the 1 M stock, making 1× dilution stocks in 2× SDC. To start the experiment, 200 µl of each 5× stock was added to 800 µl cells in the 96 well plates at time 0. The cells were incubated and shook at 30°C and were sampled every 30 min by 12-channel pipetting 75 µl of each culture into a 96 well microtiter plate. 5 µl of each 75 µl was subjected to flow cytometry analysis using a BD LSR-II equipped with a high throughput sampler, a 488 nm 100 mW laser, FITC emission filter, and FACS DIVA software to compile .fcs files. .fcs files were analyzed in MatLab and/or FloJo. No cuts or gates were applied to cell distributions. Median FITC-A values were calculated for each dose-time point and plotted in ProFit. Errors are calculated from the standard deviation of the median for 3 biological replicates.

### Ire1 FRET Assay and Confocal Microscopy

We constructed the experimental FRET strain by co-expressing Ire1-GFP and Ire1-mCherry in the same cell from the endogenous *IRE1* promoter integrated in the genome of an *Ire1*Δ strain and constructed bleed-through control strains by expressing either Ire1-GFP or Ire1-mCherry integrated alone in the deletion strain. FRET assays were performed using a Yokogawa CSU-22 spinning disc confocal on a Nikon TE-2000 inverted microscope equipped with 150 mW 488 and 562 nm lasers. Cells bearing the reporters were grown in 2× SDC to mid log phase, diluted to OD_600_ = 0.1, gently sonicated, and 80 µl added to 96 well glass bottom plates coated with concanavalin-A. Cells were allowed to settle for 20 min before imaging. DTT dilutions were prepared as 5× working stocks as in the titration time course experiments, and 20 µl added to wells at time 0.

Cells were imaged at each time point with 3×3 s exposures: 488 excitation/590 emission (GCh), 562 ex/590 em (ChCh), 488 ex/520 em (GG). Images were processed by first identifying cell boundaries and assigning the 16-bit fluorescence images to individual cells using the open-source cell-id software. Background was calculated by the mean intensity of areas in each fluorescent image not assigned to cells and subtracted from the cellular mean intensities to obtain corrected single cell values for GG, ChCh, and GCh.

The GCh value is a conglomerate of true FRET signal and fluorescent channel bleed-through from the individual fluorophores. The average GCh values from the single-fluorophore control strains were subtracted from the experimental strain GCh values to obtain final corrected values. FRET was calculated for each cell with the formula: F = GCh/(GG*ChCh)∧0.5.

For each time point at each dose, we obtained images of three different fields of cells, collecting a total of 30–60 cells per dose per time point. Mean values were plotted in ProFit and error bars represent the standard error of the mean.

### Immunoprecipitation and Western Blot Analysis

Cells bearing C-terminally HA-tagged Ire1 or Ire1^bipless^ expressed from the *IRE1* promoter on 2 micron plasmids were cultured, collected, and stored in the same manner as for the Northern blot analysis. Cell pellets were thawed on ice, resuspended in 1 ml IP buffer (50 mM Tris-HCl, pH 8.0, 150 mM NaCl, 1% Triton X-100, protease inhibitors), and subjected to bead-beating (5×1 min, with 2 min on ice between iterations). Beads and cell debris were centrifuged and the cell free lysate was incubated with anti-HA conjugated agarose beads for 2 h at 4°C. Beads were spun, washed 5× with 1 ml IP buffer, and boiled in SDS-PAGE loading buffer.

Samples were run on BioRad ready-gels (4%–15% acrylamide, Tris/glycine/SDS) for 90 min at 35 mA. The proteins were subsequently transferred to Millipore Immobilon PVDF membranes at 220 mA for 45 min. Blots were blocked in 1% casein in TBS (10 mM Tris, 150 mM NaCl) for 30 min, followed by incubation with primary antibodies overnight. The rabbit polyclonal anti-Kar2 was used at 1∶5000 dilution, and the mouse anti-HA was used at 1∶2000. The next morning, the blots were washed 3× for 10 min with TBS, and then incubated with Li-Cor fluorescently-coupled secondary antibodies, goat anti-mouse 680 and 800, at 1∶10,000 dilution for 30 min. Blots were again washed 3× for 10 min with TBS, scanned with the Li-Cor infrared scanner, and processed with the Odyssey software package.

### DTT Washout Experiments

Wild type and Ire1^bipless^ were cultured to OD_600_ = 0.4 in 400 ml 2×SDC at 30°. Cultures were brought to 500 ml and treated with 5 mM DTT for 1 h. Cells were sampled, filtered onto nitrocellulose membranes with 1 µm pores, washed with 100 ml 2×SDC, and then resuspended in 500 ml 2×SDC and returned to 30° incubation and sampled as indicated. For the FRET washout experiment, 1 ml cultures were spun, washed, resuspended, and imaged.

## Supporting Information

Figure S1 **Cell growth and UPR target gene expression in the absence of inositol.** (A) Inositol was depleted from a yeast culture and growth was monitored over time by optical density. Compared to a logarithmically growing control strain, cells depleted of inositol display a transient growth lag followed by a return to exponential growth. (B) Expression of the TR (see text) measured over time following inositol depletion. The reporter fluorescence continues to increase after the splicing of *HAC1* mRNA has returned to baseline (Figure 1A) and remains elevated.
(0.23 MB TIF)

Figure S2 **Northern blot time courses of** ***HAC1*** **mRNA in cells treated with (A) 1.5, (B) 2.2, and (C) 5 mM DTT.**
(0.31 MB TIF)

Figure S3 **Titration time course of** ***ERO1*** **promoter driving expression of GFP.** Cells bearing chromosomally integrated *pERO1-GFP* were treated with various doses of DTT and measured over time by flow cytometry. The response from the *ERO1* promoter closely matches the TR and SR.
(0.16 MB TIF)

Figure S4 **Cell-to-cell variation of Ire1^bipless^.** (A) 20 images of individual cells bearing wild type GFP tagged Ire1. The signal is homogenously distributed in the ER. (B) 20 images of individual cells bearing GFP tagged Ire1^bipless^. The signal is diffused in the ER in some cells and clustered to varying degrees in other cells. This increased variation compared to the wild type may indicate that low levels of *HAC1* mRNA splicing may occur in the absence of ER stress, but that this is below the limit of detection by Northern blot once the population has been averaged.
(1.58 MB TIF)

Figure S5 **Absolute SR fluorescence in wild type, Δ** ***hac1*** **, and Ire1^bipless^ cells.** Median values of SR fluorescence in unstressed (−) and cells treated with 5 mM DTT for 3 h (+). Error bars represent the standard deviation of three experiments.
(0.11 MB TIF)

Figure S6 **A single cell reporter of the splicing reaction.** (A) Schematic of the splicing reporter (SR) depicting the unspliced mRNA. The SR consists of a GFP-encoding exon, and the intron, splice sites, and untranslated regions identical to the *HAC1* mRNA. (B) Expression of the SR from the *HAC1* promoter does not compete with the endogenous *HAC1* mRNA for Ire1 under ER stress conditions.
(0.12 MB DOC)

Figure S7 **Rates of SR production across DTT titration time courses.** (A) Wild type cells show transient activation at 1.5 and 2.2 mM. (B) *hac1*Δ cells are fully activated until the reporter saturates at all doses. (C) Ire1^bipless^ cells are fully activated at 1.5 and 2.2 mM DTT, and show transient activation at 0.66 and 0.99 mM DTT.
(0.29 MB TIF)

Figure S8 **Target gene induction function.** (A) Function describing the transcriptional induction of UPR target genes, like for most other model parameters, was fit to experimental data found in the literature.
(0.30 MB TIF)

Figure S9 **Nonlinearity is required to recapitulate the difference between wild type and Ire1^bipless^ cells in a computational model of the UPR.** (A) Simulated DTT dose response of “wild type,” “*hac1*Δ,” and “Ire1^bipless^” models including a nonlinear term describing the dissociation of the active Ire1-unfolded protein complex. The hypersensitivity of *hac1*Δ and the intermediate sensitivity of Ire1^bipless^ are recapitulated. (B) Simulated washout experiment including nonlinearity matches experimental data. (C) Simulated DTT dose response of “wild type,” “*hac1*Δ,” and “Ire1^bipless^” models including only linear terms. No significant difference between wild type and Ire1^bipless^ is predicted. (D) Simulated washout experiment with all linear terms does not recapitulate the experimental results.
(0.55 MB TIF)

Figure S10 **Heuristic model for the nonlinearity of Ire1 deactivation.** (A) Top-down view of an active Ire1 oligomer. The molecules in the middle of the oligomer do not have the chance to dissociate from the oligomer and are hence kinetically trapped in the active mode. This results in the cooperative deactivation of active Ire1 complexes. (B) The deactivation function of the active Ire1-unfolded protein complex is a nonlinear hill function of the concentration of the active complex.
(0.32 MB TIF)

Figure S11 **Model predictions are robust to variation of floating parameters.** Sensitivity of model results to parameter variations about the best fit (solid curves). Simulations of the washout experiment were run over a range of parameter. Results are shown for three. Black curves are wild type, and green curves are Ire1^bipless^. (A) S_u_ is source (rate of UP import). (B) a_up_ is ratio of affinities of Ire1 and BiP for unfolded proteins. (C) R is affinity of BiP for free Ire1.
(0.40 MB TIF)

Figure S12 **Model predictions are robust to variation in literature-derived parameters.** (A) In silico dose responses of “wild type,” “*hac1*Δ,” and “Ire1^bipless^" models with the folding time (S_u) varied. The dose response simulations are robust to changes in the folding time of proteins in the ER. (B) The deactivation delay of Ire1^bipless^ following simulated washout is robust to changes in folding time (S_u) of proteins in the ER. (C) The deactivation delay of Ire1^bipless^ following simulated DTT washout is robust to changes in the cellular diffusion constant. (D) Variation in the number of Ire1 molecules should affect the deactivation kinetics of Ire1^bipless^ more than wild type.
(0.63 MB TIF)

Figure S13 **BiP re-associates with Ire1 with kinetics that match Ire1 deactivation following DTT washout.** (A) Cells bearing HA-tagged, wild type Ire1 were treated with 5 mM DTT for 1 h. DTT was washed by filtration and cells were collected over time. Ire1 was immuno-precipitated from lysates, and precipitates were immuno-blotted with antibodies against Ire1 (anti-HA) and BiP (anti-Kar2) (see Methods). (B) The ratio of BiP to Ire1 in each lane above. BiP re-associates with Ire1 to the level of unstressed cells.
(0.89 MB TIF)

Figure S14 **Characterization and quantification of Ire1 FRET reporter.** (A) Expression of the FRET reporter allows cells to splice *HAC1* mRNA as well as wild type. (B) Images of Ire1-GFP, Ire1-mCherry, and raw Ire1 FRET from unstressed cells and cells treated with 5 mM DTT for 180 min. (C) Example images of fluorescence bleed through images in stressed and unstressed cells. Bleed through was subtracted from the raw FRET signal as a function of dose and time. (D). Single cells were defined and FRET from single cells was quantified using Cell ID 1.4 [27].
(2.55 MB TIF)

Figure S15 **Ire1^bipless^ cells display increased cell-to-cell variation in the absence of stress.** Histograms of wild type and Ire1^bipless^ cells expressing the splicing and transcriptional reporters in the absence of stress. Different color histograms represent separate experiments. Inset number are the standard deviation divided by the mean (CV). Ire1^bipless^ cells have increased variation compared to the wild type despite the increased mean.
(0.50 MB TIF)

Text S1 **Computational model and methods.**
(0.12 MB PDF)

## Abbreviations

DTT: dithiothreitol
ER: endoplasmic reticulum
FRET: fluorescence resonance energy transfer
GFP: green fluorescent protein
PDI: protein disulfide isomerase
SR: splicing reporter
TR: transcriptional reporter
UPR: unfolded protein response
