# Supplementary figures and images for: BiP Binding to the ER-Stress Sensor Ire1 Tunes the Homeostatic Behavior of the Unfolded Protein Response
Source: PLoS Biol. 2010 Jul 6;8(7):e1000415. doi: 10.1371/journal.pbio.1000415 (PMC2897766; doi:10.1371/journal.pbio.1000415)

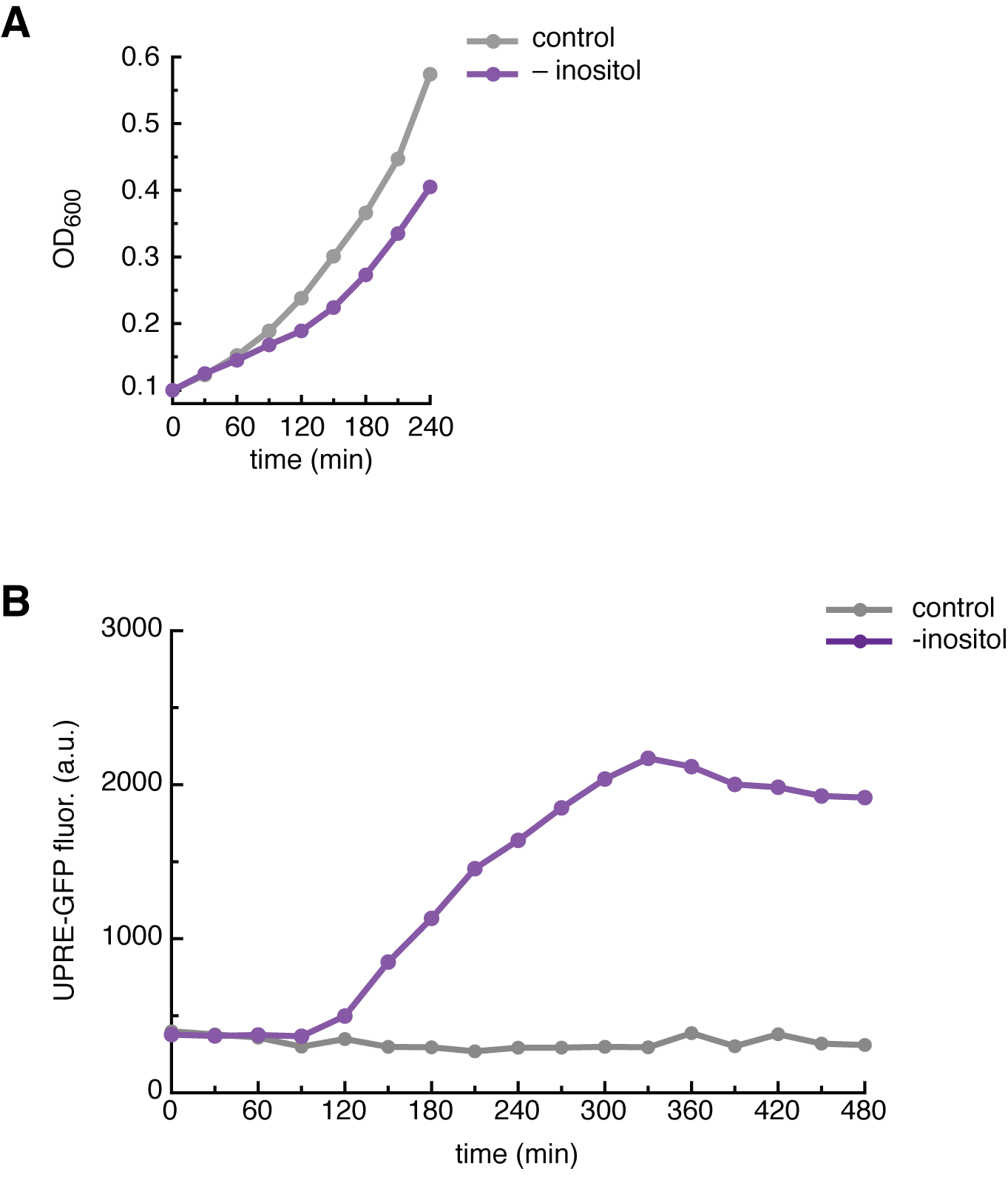

Supplement: Figure S1 — Cell growth and UPR target gene expression in the absence of inositol. (A) Inositol was depleted from a yeast culture and growth was monitored over time by optical density. Compared to a logarithmically growing control strain, cells depleted of inositol display a transient growth lag followed by a return to exponential growth. (B) Expression of the TR (see text) measured over time following inositol depletion. The reporter fluorescence continues to increase after the splicing of HAC1 mRNA has returned to baseline (Figure 1A) and remains elevated. (0.23 MB TIF) [file pbio.1000415.s001.tif]

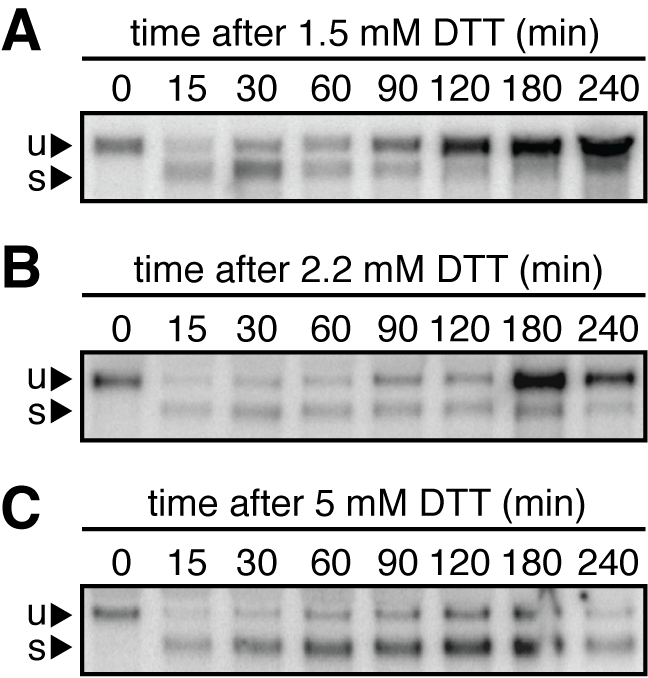

Supplement: Figure S2 — Northern blot time courses of HAC1 mRNA in cells treated with (A) 1.5, (B) 2.2, and (C) 5 mM DTT. (0.31 MB TIF) [file pbio.1000415.s002.tif]

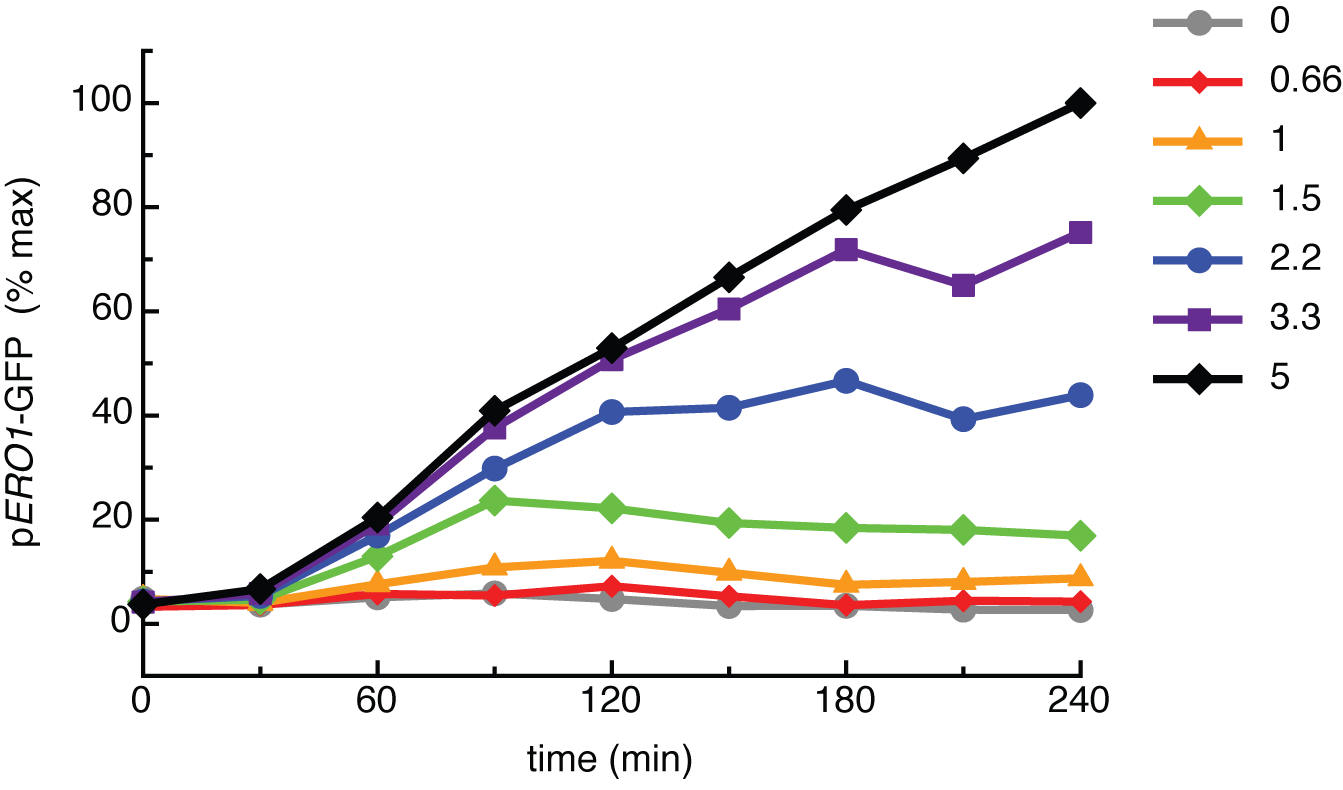

Supplement: Figure S3 — Titration time course of ERO1 promoter driving expression of GFP. Cells bearing chromosomally integrated pERO1-GFP were treated with various doses of DTT and measured over time by flow cytometry. The response from the ERO1 promoter closely matches the TR and SR. (0.16 MB TIF) [file pbio.1000415.s003.tif]

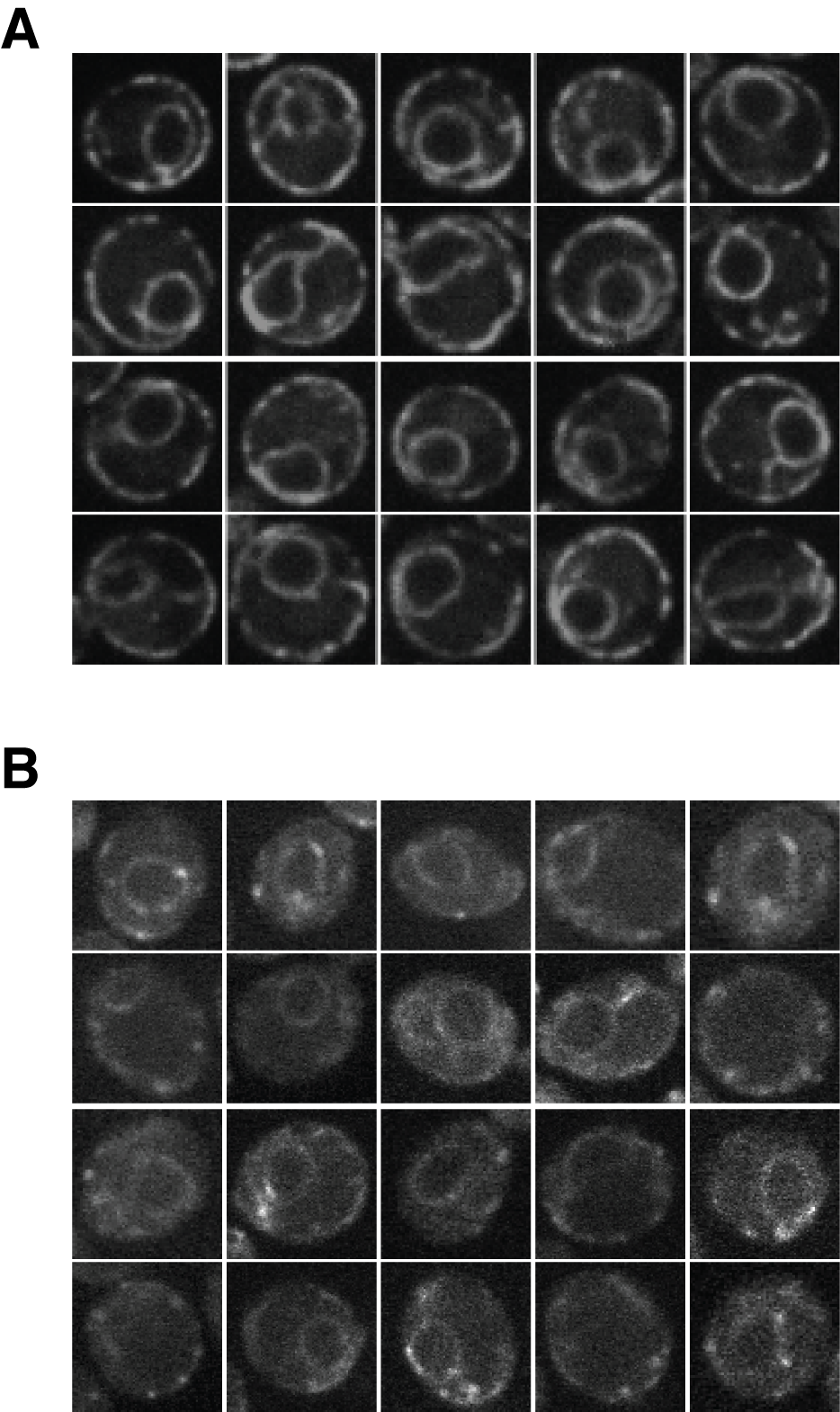

Supplement: Figure S4 — Cell-to-cell variation of Ire1bipless. (A) 20 images of individual cells bearing wild type GFP tagged Ire1. The signal is homogenously distributed in the ER. (B) 20 images of individual cells bearing GFP tagged Ire1bipless. The signal is diffused in the ER in some cells and clustered to varying degrees in other cells. This increased variation compared to the wild type may indicate that low levels of HAC1 mRNA splicing may occur in the absence of ER stress, but that this is below the limit of detection by Northern blot once the population has been averaged. (1.58 MB TIF) [file pbio.1000415.s004.tif]

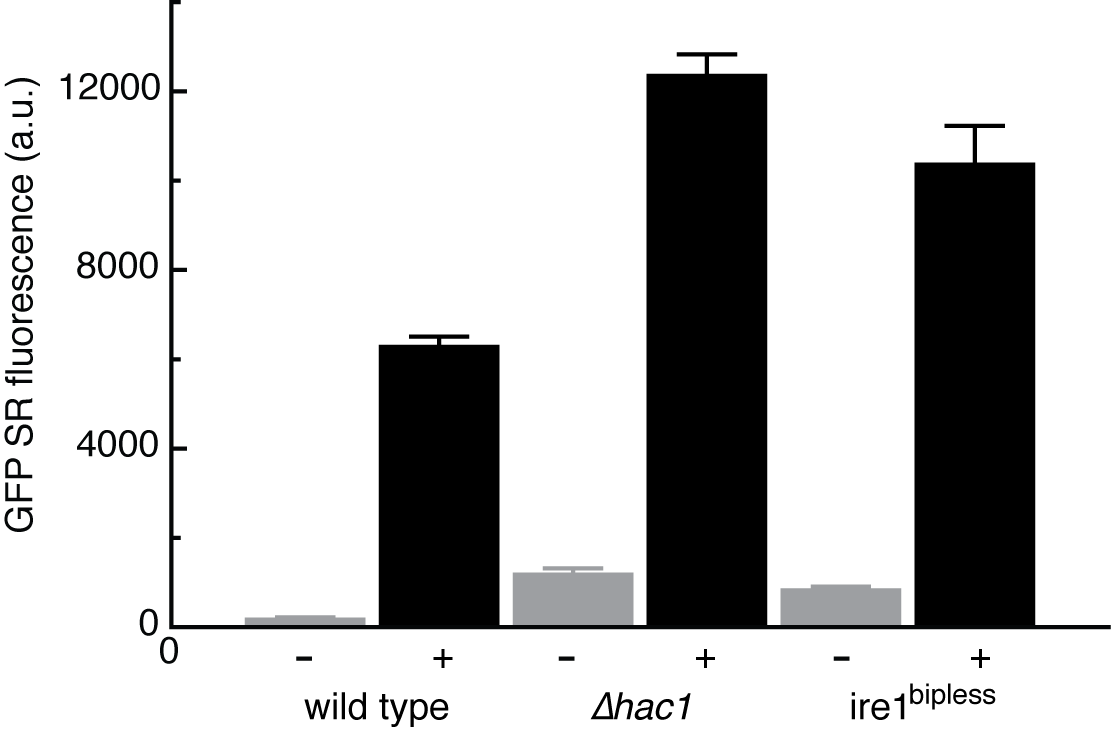

Supplement: Figure S5 — Absolute SR fluorescence in wild type, Δ hac1 , and Ire1bipless cells. Median values of SR fluorescence in unstressed (−) and cells treated with 5 mM DTT for 3 h (+). Error bars represent the standard deviation of three experiments. (0.11 MB TIF) [file pbio.1000415.s005.tif]

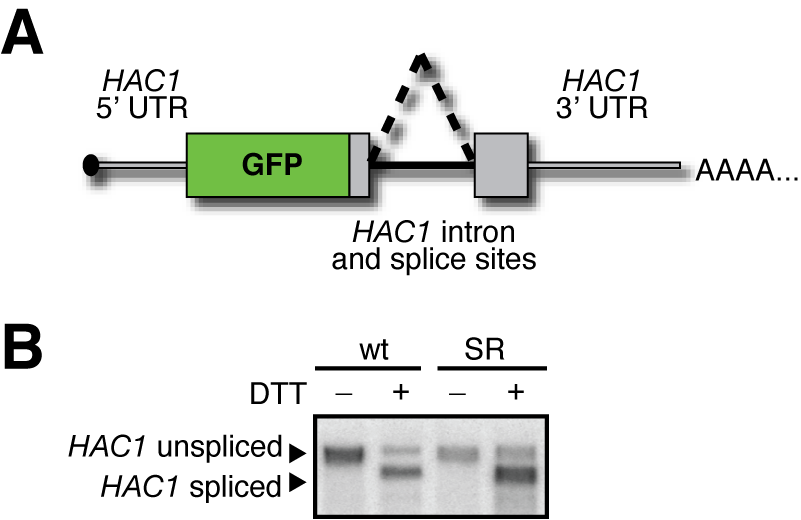

Supplement: Figure S6 — A single cell reporter of the splicing reaction. (A) Schematic of the splicing reporter (SR) depicting the unspliced mRNA. The SR consists of a GFP-encoding exon, and the intron, splice sites, and untranslated regions identical to the HAC1 mRNA. (B) Expression of the SR from the HAC1 promoter does not compete with the endogenous HAC1 mRNA for Ire1 under ER stress conditions. (0.12 MB DOC) [file pbio.1000415.s006.tif]

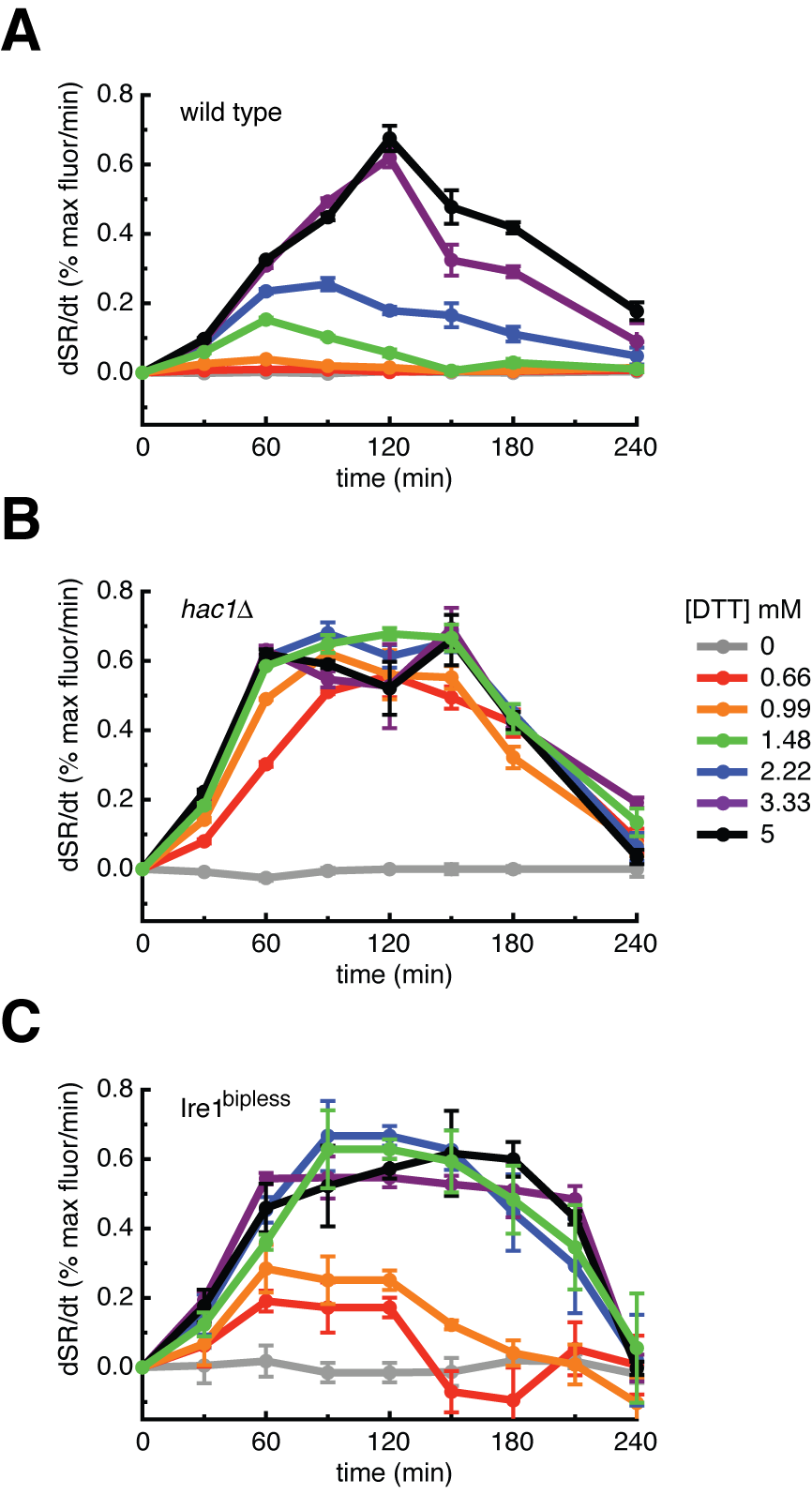

Supplement: Figure S7 — Rates of SR production across DTT titration time courses. (A) Wild type cells show transient activation at 1.5 and 2.2 mM. (B) hac1Δ cells are fully activated until the reporter saturates at all doses. (C) Ire1bipless cells are fully activated at 1.5 and 2.2 mM DTT, and show transient activation at 0.66 and 0.99 mM DTT. (0.29 MB TIF) [file pbio.1000415.s007.tif]

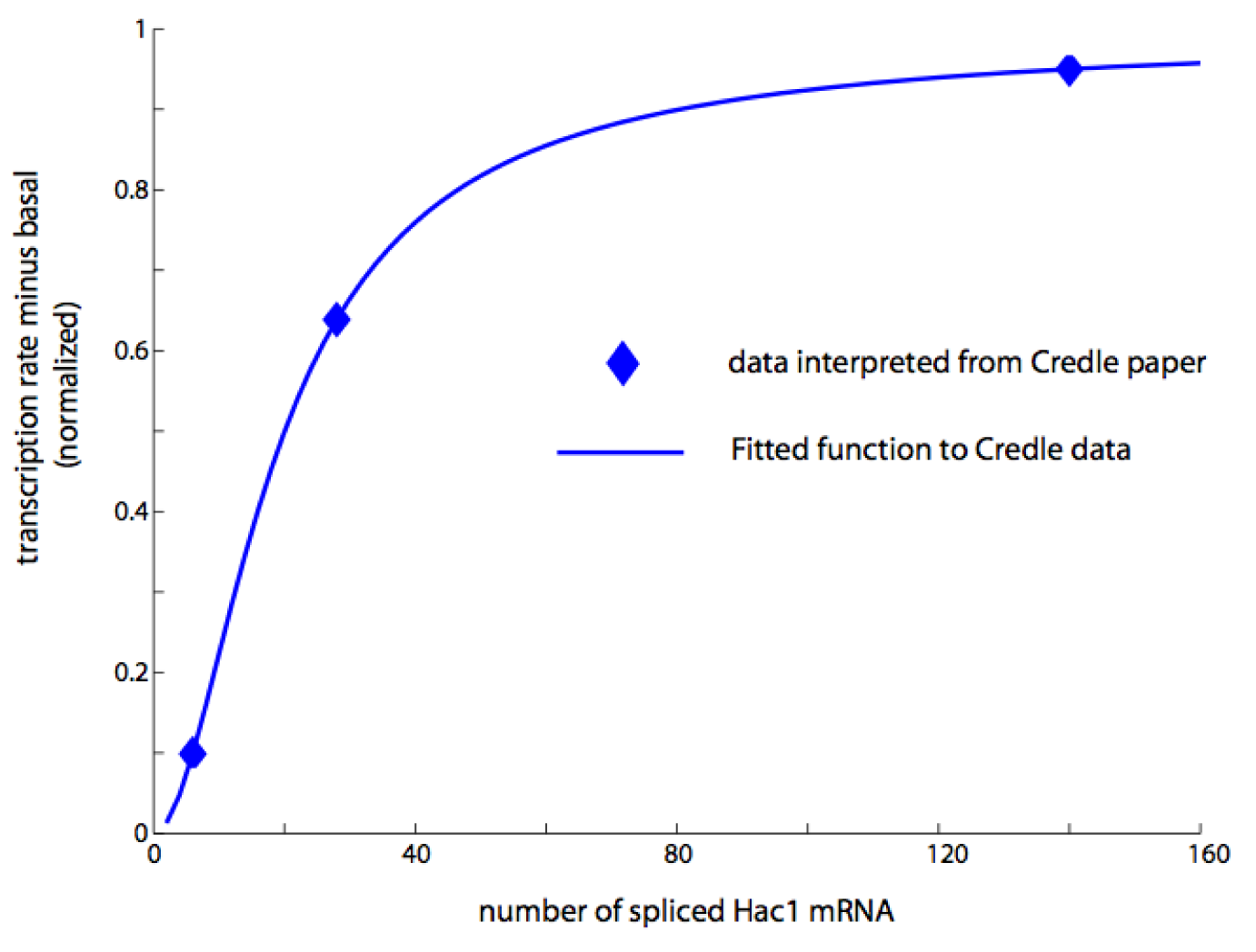

Supplement: Figure S8 — Target gene induction function. (A) Function describing the transcriptional induction of UPR target genes, like for most other model parameters, was fit to experimental data found in the literature. (0.30 MB TIF) [file pbio.1000415.s008.tif]

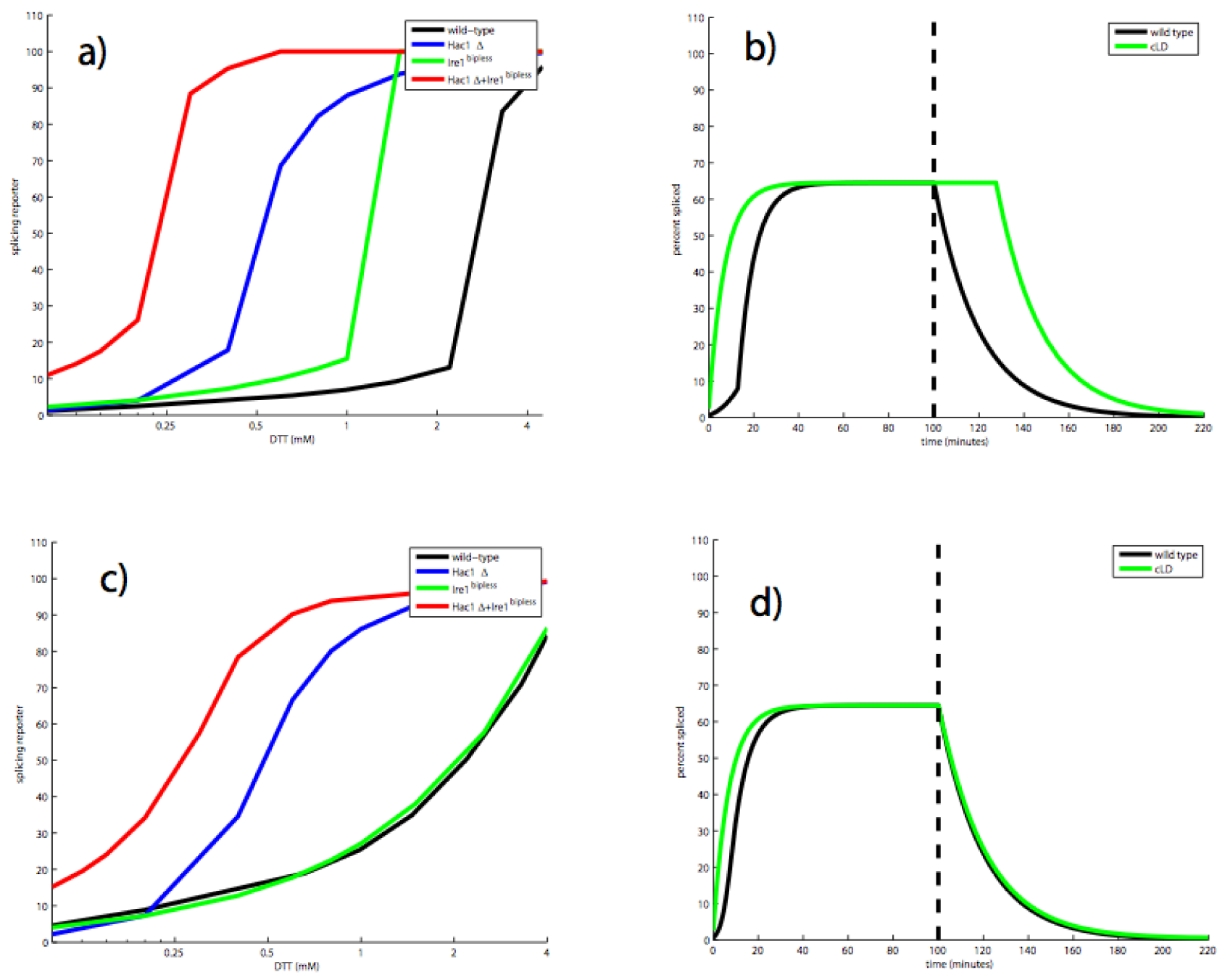

Supplement: Figure S9 — Nonlinearity is required to recapitulate the difference between wild type and Ire1bipless cells in a computational model of the UPR. (A) Simulated DTT dose response of “wild type,” “hac1Δ,” and “Ire1bipless” models including a nonlinear term describing the dissociation of the active Ire1-unfolded protein complex. The hypersensitivity of hac1Δ and the intermediate sensitivity of Ire1bipless are recapitulated. (B) Simulated washout experiment including nonlinearity matches experimental data. (C) Simulated DTT dose response of “wild type,” “hac1Δ,” and “Ire1bipless” models including only linear terms. No significant difference between wild type and Ire1bipless is predicted. (D) Simulated washout experiment with all linear terms does not recapitulate the experimental results. (0.55 MB TIF) [file pbio.1000415.s009.tif]

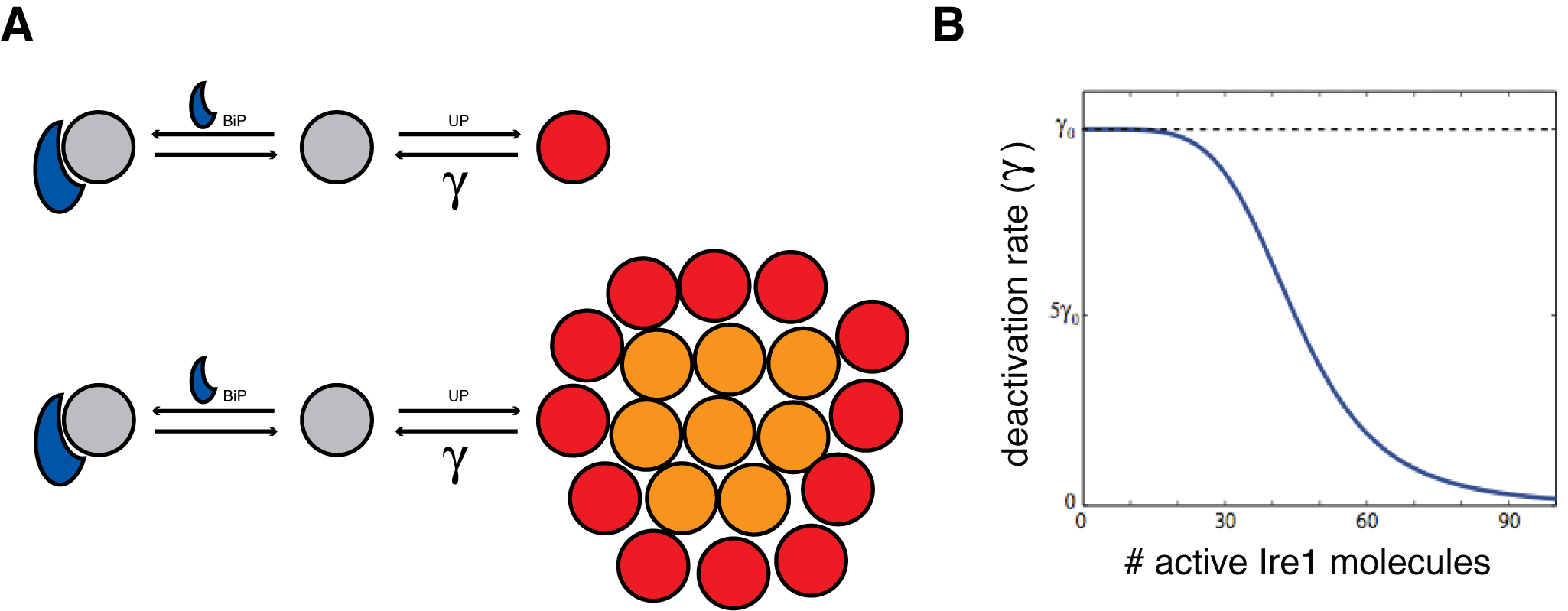

Supplement: Figure S10 — Heuristic model for the nonlinearity of Ire1 deactivation. (A) Top-down view of an active Ire1 oligomer. The molecules in the middle of the oligomer do not have the chance to dissociate from the oligomer and are hence kinetically trapped in the active mode. This results in the cooperative deactivation of active Ire1 complexes. (B) The deactivation function of the active Ire1-unfolded protein complex is a nonlinear hill function of the concentration of the active complex. (0.32 MB TIF) [file pbio.1000415.s010.tif]

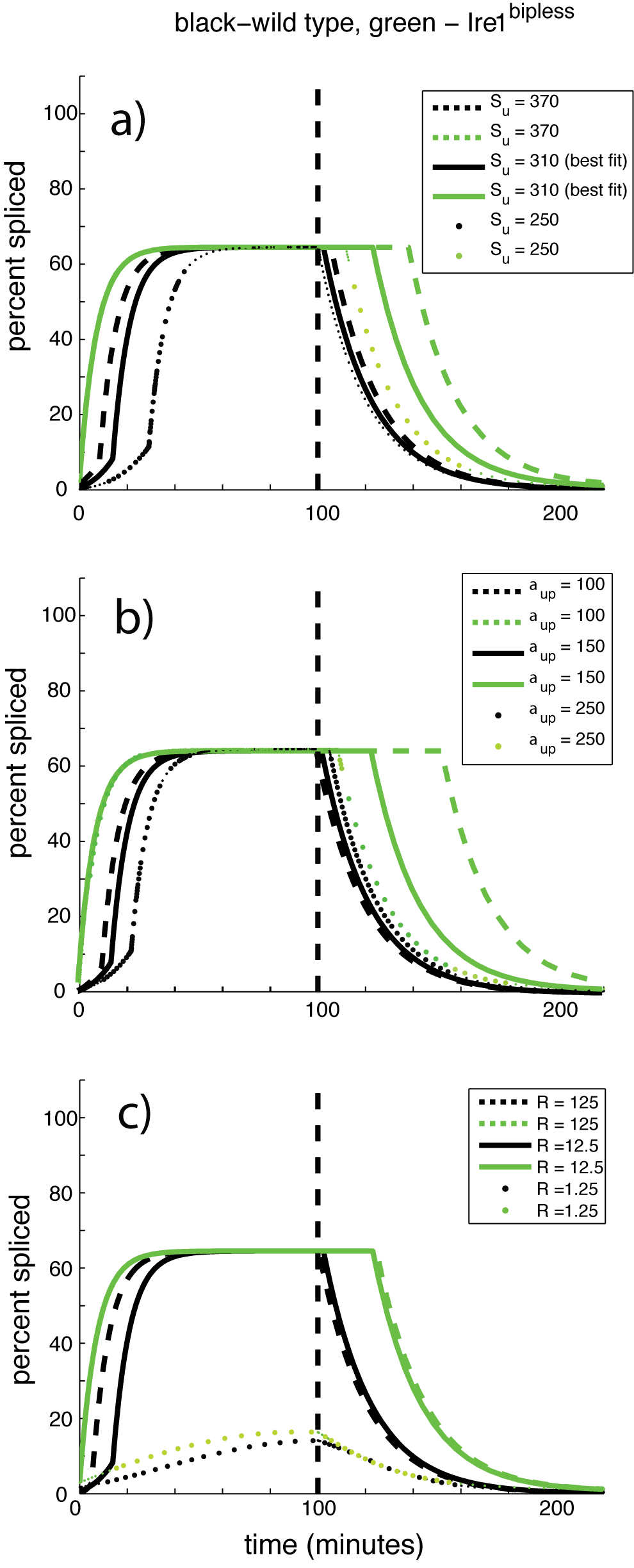

Supplement: Figure S11 — Model predictions are robust to variation of floating parameters. Sensitivity of model results to parameter variations about the best fit (solid curves). Simulations of the washout experiment were run over a range of parameter. Results are shown for three. Black curves are wild type, and green curves are Ire1bipless. (A) Su is source (rate of UP import). (B) aup is ratio of affinities of Ire1 and BiP for unfolded proteins. (C) R is affinity of BiP for free Ire1. (0.40 MB TIF) [file pbio.1000415.s011.tif]

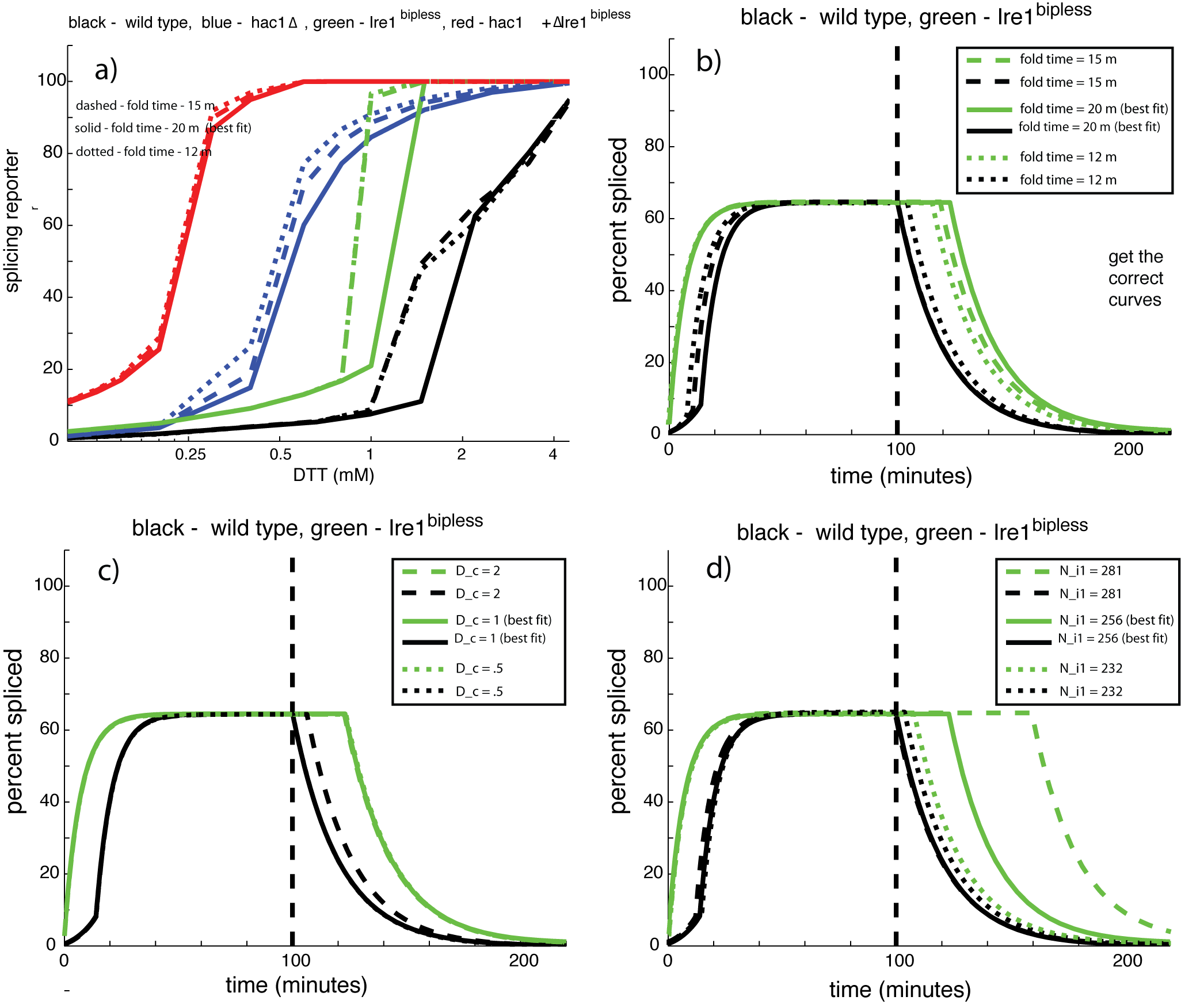

Supplement: Figure S12 — Model predictions are robust to variation in literature-derived parameters. (A) In silico dose responses of “wild type,” “hac1Δ,” and “Ire1bipless" models with the folding time (S_u) varied. The dose response simulations are robust to changes in the folding time of proteins in the ER. (B) The deactivation delay of Ire1bipless following simulated washout is robust to changes in folding time (S_u) of proteins in the ER. (C) The deactivation delay of Ire1bipless following simulated DTT washout is robust to changes in the cellular diffusion constant. (D) Variation in the number of Ire1 molecules should affect the deactivation kinetics of Ire1bipless more than wild type. (0.63 MB TIF) [file pbio.1000415.s012.tif]

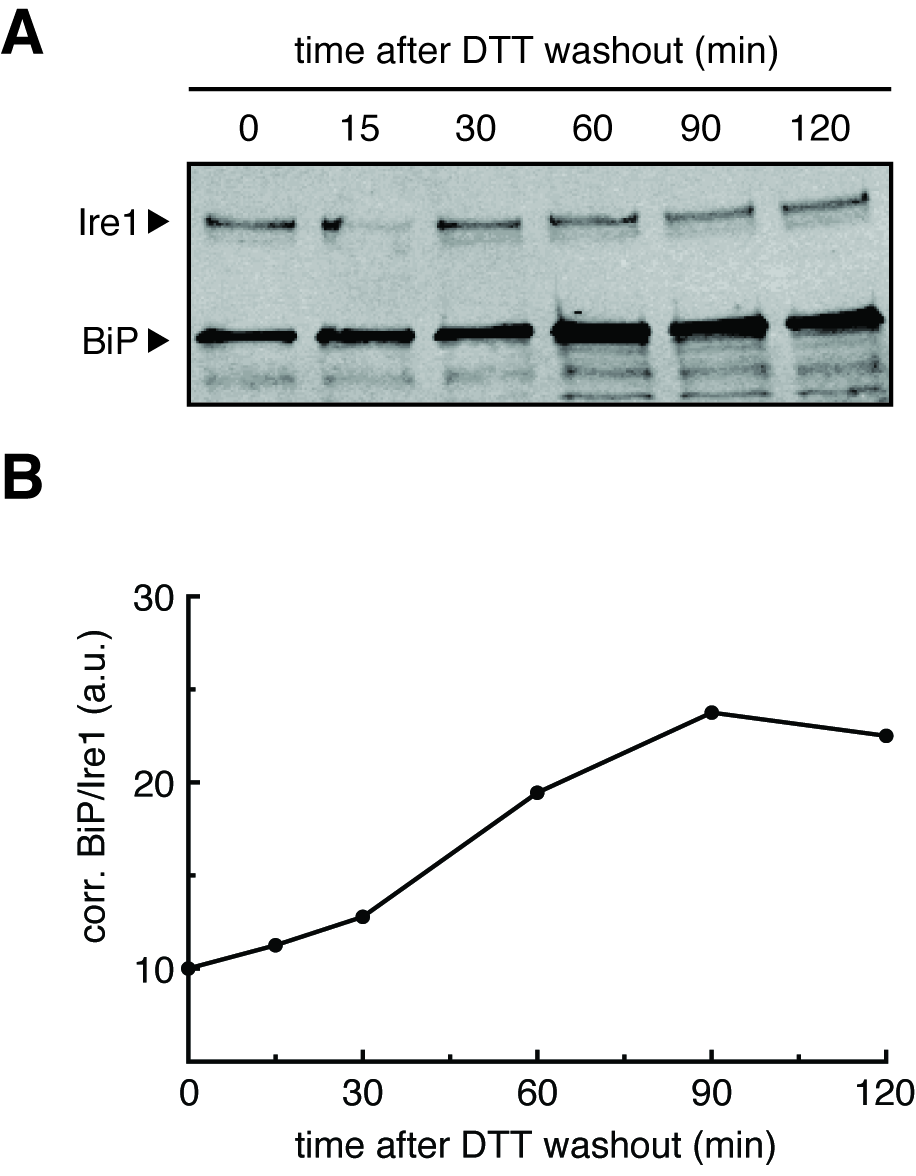

Supplement: Figure S13 — BiP re-associates with Ire1 with kinetics that match Ire1 deactivation following DTT washout. (A) Cells bearing HA-tagged, wild type Ire1 were treated with 5 mM DTT for 1 h. DTT was washed by filtration and cells were collected over time. Ire1 was immuno-precipitated from lysates, and precipitates were immuno-blotted with antibodies against Ire1 (anti-HA) and BiP (anti-Kar2) (see Methods). (B) The ratio of BiP to Ire1 in each lane above. BiP re-associates with Ire1 to the level of unstressed cells. (0.89 MB TIF) [file pbio.1000415.s013.tif]

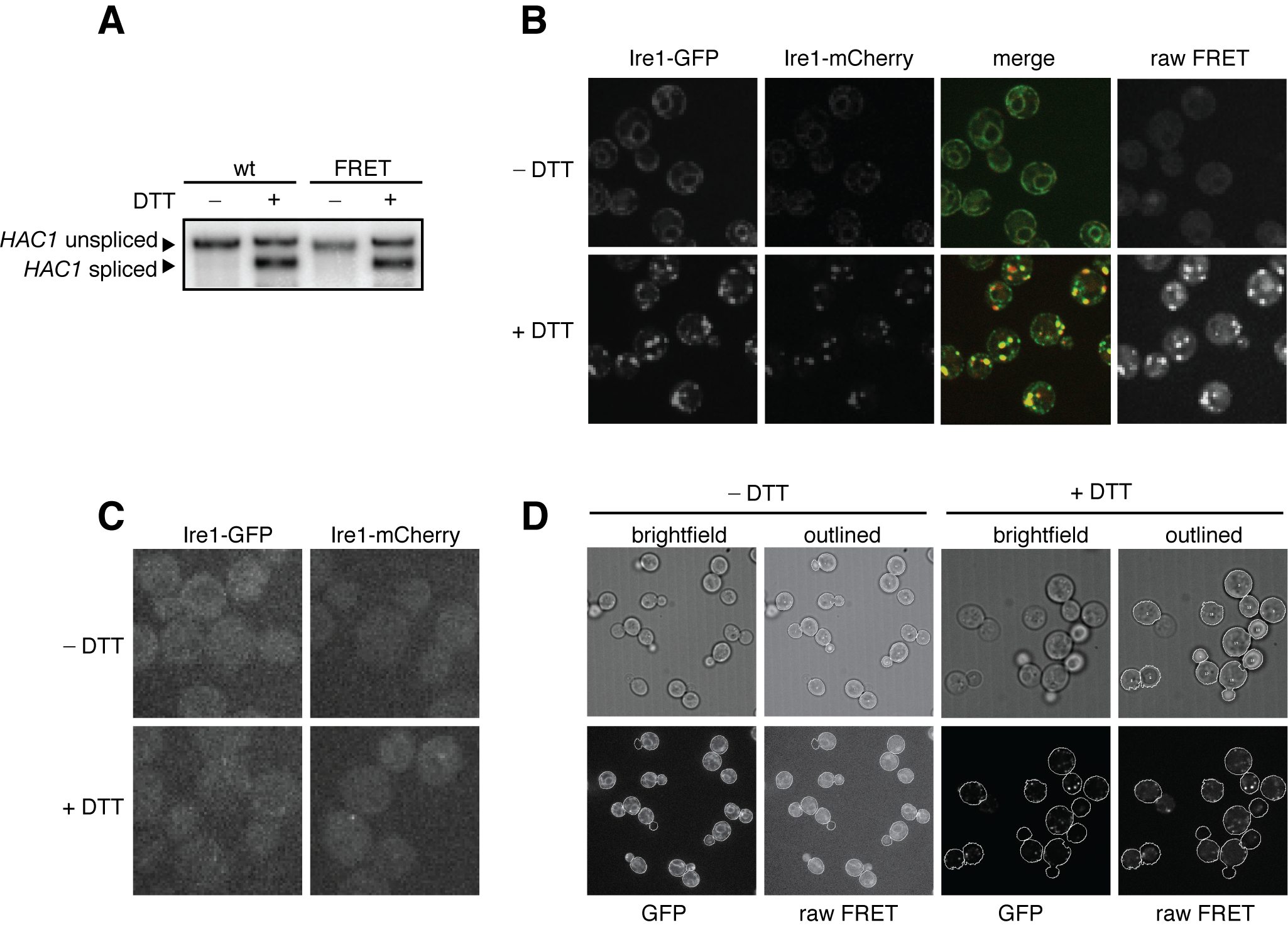

Supplement: Figure S14 — Characterization and quantification of Ire1 FRET reporter. (A) Expression of the FRET reporter allows cells to splice HAC1 mRNA as well as wild type. (B) Images of Ire1-GFP, Ire1-mCherry, and raw Ire1 FRET from unstressed cells and cells treated with 5 mM DTT for 180 min. (C) Example images of fluorescence bleed through images in stressed and unstressed cells. Bleed through was subtracted from the raw FRET signal as a function of dose and time. (D). Single cells were defined and FRET from single cells was quantified using Cell ID 1.4 [27]. (2.55 MB TIF) [file pbio.1000415.s014.tif]

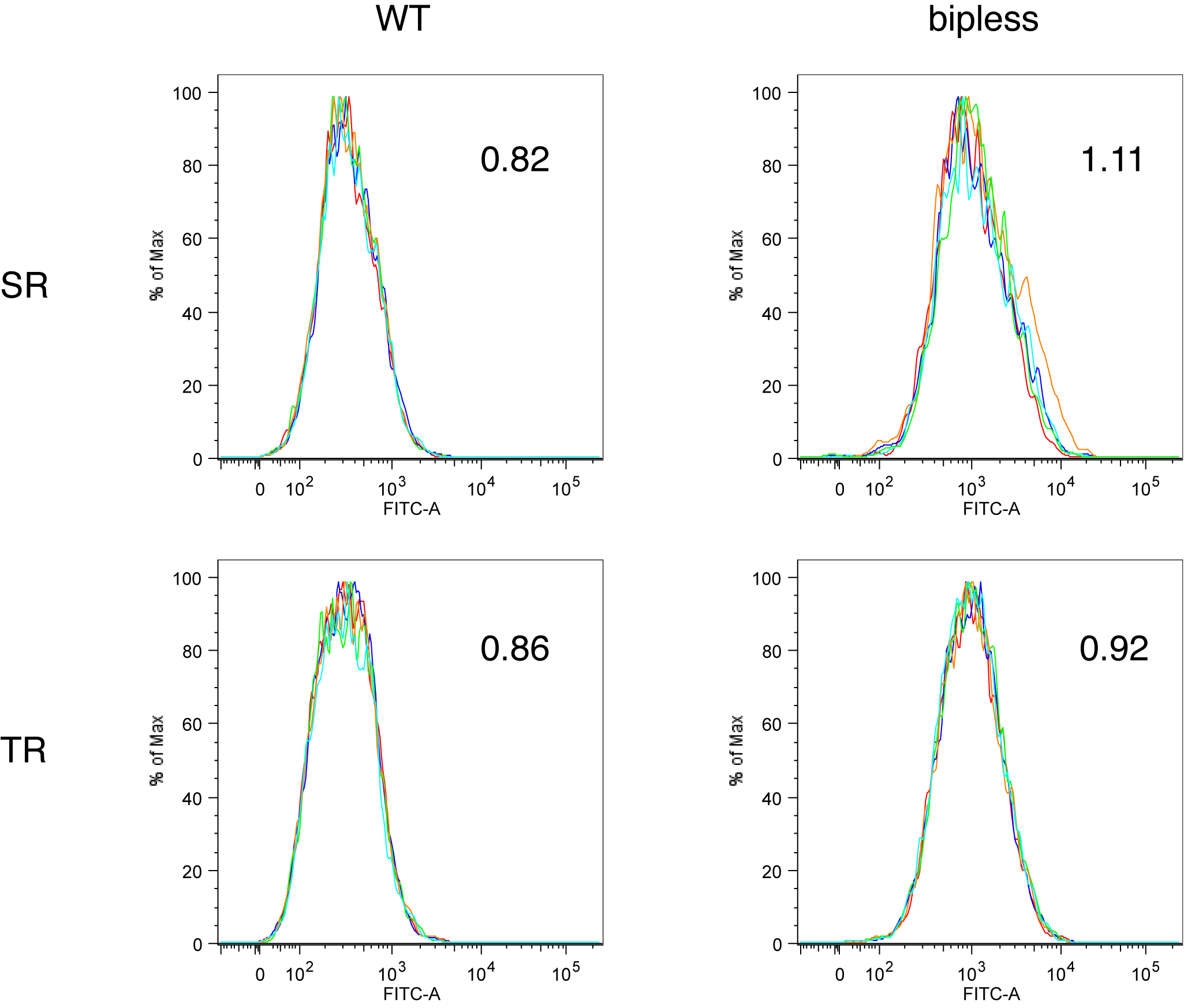

Supplement: Figure S15 — Ire1bipless cells display increased cell-to-cell variation in the absence of stress. Histograms of wild type and Ire1bipless cells expressing the splicing and transcriptional reporters in the absence of stress. Different color histograms represent separate experiments. Inset number are the standard deviation divided by the mean (CV). Ire1bipless cells have increased variation compared to the wild type despite the increased mean. (0.50 MB TIF) [file pbio.1000415.s015.tif]
