# Supplementary material for: BiP Binding to the ER-Stress Sensor Ire1 Tunes the Homeostatic Behavior of the Unfolded Protein Response
Source: PLoS Biol. 2010 Jul 6;8(7):e1000415. doi: 10.1371/journal.pbio.1000415 (PMC2897766; doi:10.1371/journal.pbio.1000415)
Supplement: Text S1 — Computational model and methods. (0.12 MB PDF) [file pbio.1000415.s016.pdf]

Computational Model

In this section, we present the computational model developed to capture the salient features of the UPR dynamics. The model we build is based on deterministic mass-action kinetics, resulting in a set of ordinary differential equations.

1 Model Variables

The variables in the model describe the number of molecules rather than concentrations. For ease of notation and clarity of model equations, we will put square brackets around each variable, e.g. $[X]$. A description of the model variables is given in the table below.

variable	description
B_m	BiP messenger RNA
B	BiP protein
E_{1_m}	Ero1 messenger RNA
E_1	Ero1 protein, stabilizes REDOX potential
D_0	dose level of DTT, increases REDOX potential
I_1	Ire1 monomer
$I_1 \cdot B$	Ire1 bound to BiP, inactive Ire1
I_{1A}	Ire1 bound to an unfolded protein, active Ire1,
$H^u_{1_m}$	Unspliced Hac1 messenger RNA
$H^s_{1_m}$	Spliced Hac1 messenger RNA
U	Unfolded protein
$U \cdot B$	folding complex (folding protein with/chaperone)
U_d	Unfolded proteins with broken disulfide bonds
$U_d \cdot B$	misfolded complex (folding protein with broken disulfide bonds/chaperone)

2 Model Assumptions

In building the computational model, we adopted the following biologically motivated assumptions

- Ire1 is activated through contact with unfolded protein that are not in the process of folding properly, that is U, U_d, and $U_d \cdot B$. It is not activated by $U \cdot B$ which is folding properly.
- Only activated Ire1 splices Hac1.
- BiP titrates unfolded proteins, preventing unfolding proteins from interacting with Ire1.
- DTT breaks disulfide bonds in the unfolded protein and folding complexes and this process occurs as an enzymatic reaction. We also assume that there is a constant pool of DTT diffusing in and out of the ER, replenishing the local population.
- Ero1 mends broken disulfide bonds resulting from DTT. The population of unfolded proteins with broken disulfide bonds vs. those without is dependent on the relative populations of Ero1

vs. DTT. In reality, Ero1 is one protein of many that assist in the formation of disulfide bonds, others include oxidoreductases from the PDI family. For simplicity, Ero1 will represent the entire mechanism for disulfide bond formation and will act as an enzyme.

- Ero1 and DTT are equally efficient in their enzymatic reactions. Therefore the competition is 1-to-1.
- Unfolded proteins regulate an allosteric switch in Ire1 from the inactive state to the active state. Ire1 then allosterically deactivates based on the active state's deactivation rate. This model is chosen over the alternate model that unfolded protein activate Ire1 by binding to it and deactivate by dissociating from it. Mathematically, the allosteric model is simpler and because of the small number of Ire1 and the generally large number of unfolded proteins, either scenario would yield essentially the same results.
- The deactivation rate of active Ire1 is non-linearly dependent on the population of active Ire1. The relationship we use is pictorially described in Figure S10a. Ire1 molecules are known to form foci which are correlated with Hac1 mRNA splicing. The nonlinear function we employ in the model is meant to serve as a simple phenomenological description of the deactivation rate of Ire1 from the Ire1 foci, formed during stress. This relationship can describe a variety of underlying mechanistic schemes of Ire1 inactivation
- The population of Ire1 is approximately 256 [*Ghaemmaghami et al.* (2003)].
- The basal population of Hac1 mRNA is 200 [*Walter*].
- The basal population of BiP is approximately 430,000 [*Ghaemmaghami et al.* (2003)].
- We are neglecting the lectin chaperone pathway.
- We are neglecting the ATP/ADP cycle of BiP which is known to switch BiP's affinity (high/ATP and low/ADP) to molecules such as Ire1 and unfolded proteins. In this model we set BiP's affinity constant and equal to both Ire1 and unfolded proteins.
- We are neglecting the contribution of ERAD.

3 Chemical Reactions and Reaction Rate Constants Used in Model

The reaction rate constants we will use in the equations are the traditional reaction rate constant divided by the volume of the space involved (area if on a membrane), e.g. our $c_\mu = k_\mu/V$ where k_μ is the rate constant for some species μ. The model consists of three modules. Below, we provide a description of the chemical reactions modeled and the reactions rates used.

3.1 Protein Folding Module

Reaction	Description
$\emptyset \xrightarrow{S_u} U$	Unfolded protein translocation into ER.
$U + B \xrightarrow{c_{[B]}} U \cdot B$	BiP attachment to unfolded protein to form folding complex.
$U \cdot B \xrightarrow{\gamma_{[U \cdot B]}} U + B$	Bip dissociation from folding complex.
$U \cdot B \xrightarrow{\gamma_{fold}} \emptyset + B$	Completion of folding and release of folded protein from folding complex.
$U + D_0 \xrightarrow{c_{D_0}} U_d$	Breakage of unfolded protein's disulfide bonds by DTT.
$U_d + E_1 \xrightarrow{c_{[E_1]}} U$	Formation of disulfide bonds by Ero1 in the unfolded protein with broken disulfide bonds.
$U_d + B \xrightarrow{c_{[B]}} U_d \cdot B$	BiP attachment to unfolded protein with broken disulfide bonds to form misfolding complex.
$U_d \cdot B \xrightarrow{\gamma_{[U \cdot B]}} U_d + B$	BiP dissociation from misfolding complex
$U \cdot B + D_0 \xrightarrow{c_{D_0}} U_d \cdot B$	Breakage of disulfide bonds in folding complex by DTT.
$U_d \cdot B + E_1 \xrightarrow{c_{[E_1]}} U \cdot B$	Ero1 forms disulfide bonds in the misfolding complex.
$U \cdot B \xrightarrow{\gamma_{[B]}} U + \emptyset$	BiP's decay from folding complex.
$U_d \cdot B \xrightarrow{\gamma_{[B]}} U_d + \emptyset$	BiP's decay from misfolding complex.

Reaction Rate Constants for Folding Module

S_u - 310 mol s^{-1}. Source rate for protein unfolding. For more details see *Crucial Parameter Fitting* section below.

$c_{[U \cdot B]}$ - .0350 mol^{-2} s^{-1}. Attachment rate of single BiP molecule. Derived using the formula $c_{[U \cdot B]} = 4\pi D_c(2a_p)/V_{er}$ [*Berg and Purcell*(1977)]. $D_c = 1$ μm^2 s^{-1} is a typical cytosolic diffusion coefficient. $a_p = .028$ μm is the typical protein size in the ER. $V_{er} = 2.15$ μm^3 is the approximate volume of the ER.

$\gamma_{[U \cdot B]}$ - 196 mol^{-1} s^{-1}. Dissociation rate of BiP from folding complex. We assume this to be equal to $\gamma_{[I_1 \cdot B]}$, the decay rate of the Ire1/BiP complexes. For more details see *Crucial Parameter Fitting* section below.

γ_{fold} - 8.33×10^{-4} mol^{-1} s^{-1}. Folding rate of protein in the folding complex. (20 minute folding time).

c_{D_0} - 1.50×10^{-3} mol^{-2} s^{-1}. Enzymatic rate of disulfide bond breaking. Value is not crucial since we are setting $c_{[E_1]} = c_{D_0}$ for 1-to-1 competition.

$c_{[E_1]}$ - 1.50×10^{-3} mol^{-2} s^{-1}. Enzymatic rate of disulfide bond formation. We have $c_{[E_1]} = c_{D_0}$ since we are assuming 1-to-1 competition.

$\gamma_{[B]}$ - 1.39×10^{-4} mol^{-1} s^{-1}, Decay rate of BiP (2 hour mean decay time). Taken from [*Axelsen and Sneppen*(2004)].

3.2 Ire1 activation, Hac1 splicing, and splicing reporter module

Reaction	Description
$I_1 + B \xrightarrow{c_{[B]}} I_1 \cdot B$	BiP binding to Ire1 to form inactive complex.
$I_1 \cdot B \xrightarrow{\gamma_{[I_1 \cdot B]}} I_1 + B$	BiP dissociation from inactive complex
$I_1 + U_g \xrightarrow{c_{up}} I_{1A} + U_g$ where $U_g = U$ or U_d or $U_d \cdot B$	Ire1 activation to enable splicing.
$I_{1A} \xrightarrow{F_{[I_{1A}]}} I_1$	Ire1 deactivation.
$\emptyset \xrightarrow{\beta_{[H^u_{1_m}]}} H^u_{1_m}$	transcription of unspliced Hac1 mRNA.
$H^u_{1_m} \xrightarrow{\gamma_{[H_{1_m}]}} \emptyset$	Decay of unspliced Hac1 mRNA.
$H^s_{1_m} \xrightarrow{\gamma_{[H_{1_m}]}} \emptyset$	Decay of spliced Hac1 mRNA.
$H^u_{1_m} \xrightarrow{\beta_{[H^s_{1_m}]}} H^s_{1_m} + R_{S_m}$	Splicing of Hac1 mRNA and reporter mRNA
$R_{S_m} \xrightarrow{\gamma_{[R_{S_m}]}} \emptyset$	Decay of splicing reporter mRNA.
$\emptyset \xrightarrow{\beta_{R_S}} R_S$	Translation of splicing reporter GFP.
$R_S \xrightarrow{\gamma_{[R_S]}} \emptyset$	Decay of splicing reporter GFP.

Reaction Rate Constants for Ire1 Activation, Hac1 Splicing, and Splicing Reporter Module

$c_{[I_1 \cdot B]}$ - .0350 mol^{-2} s^{-1}. Attachment rate of single BiP molecule to Ire1. For simplicity, we assume $c_{[I_1 \cdot B]} = c_{[U \cdot B]}$.

$\gamma_{[I_1 \cdot B]}$ - 196 mol^{-1} s^{-1}. Dissociation rate of BiP from the inactive complex. For more details see *Crucial Parameter Fitting* section below.

c_{up} - 2.33×10^{-4} mol^{-2} s^{-1}. Attachment rate of Ire1 molecule to an unfolded protein. For more details see *Crucial Parameter Fitting* section below.

$F_{[I_{1A}]}$ - $F_{[I_{1A}]} = \gamma_{[I_{1A}]} \frac{[I_0]^n}{[I_0]^n + [I_{1A}]^n}$ represents a non-linear (active Ire1 cooperative) decay rate as illustrated in Figure S10b. We have set $\gamma_{[I_{1A}]} \approx \gamma_{[I_1 \cdot B]}$ and $n = 4.5, I_0 = 45$. For more details see *Crucial Parameter Fitting* section below.

$\beta_{[H^u_{1_m}]}$ - 0.167 s^{-1} Transcription rate of Hac1 unspliced mRNA. The mean decay time of both spliced and unspliced is assumed to be 20 minutes. Therefore $\beta_{[H^u_{1_m}]}$ is set so that the steady state population of all Hac1 mRNA is equal to 200.

$\gamma_{[H_{1_m}]}$ - 8.33×10^{-4} s^{-1}. Decay rate of Hac1 mRNA corresponding to a mean decay time of 20 minutes as determined from [*Sidrauski et al.*(1996)] (Figure 5c and page 408). We use the same value for both spliced and unspliced.

$\beta_{[H^s_{1_m}]}$ - 1.5×10^{-3} mol^{-1} s^{-1}. Splicing rate of Hac1 mRNA corresponding to an 11 minute splicing time inferred from Figure 3 of [*Mori et al.*(1997)], which also shows that the total number of mHac1, spliced and unspliced remains relatively constant, an assumption in our model. The total number of molecules being spliced per second is $\beta_{[H^s_{1_m}]}\min([I_{1A}], [H^u_{1_m}])$. The function $\min([I_{1A}], [H^u_{1_m}])$ is required since the minimum of these quantities will determine the number of molecules being instantaneously spliced.

$\gamma_{[R_{S_m}]}$ 8.33×10^{-4} mol^{-1} s^{-1}. Decay rate of the splicing reporter mRNA. This is assumed to be the same as $\gamma_{[H_{1_m}]}$.

$\beta_{[R_S]}$ 8.33×10^{-3} mol^{-1} s^{-1}. Translation rate of the splicing reporter (2 minutes per translation event).

$\gamma_{[R_S]}$ 3.47×10^{-5}. Decay rate of the splicing reporter GFP (8 hour mean decay time).

3.3 BiP and Ero1 transcription module

Reaction	Description
$\emptyset \xrightarrow{F_{[B_m]}} B_m$	Transcription of BiP mRNA.
$B_m \xrightarrow{\gamma_{[B_m]}} \emptyset$	Decay of BiP mRNA.
$\emptyset \xrightarrow{\beta_{[B]}} B$	Translation of BiP.
$B \xrightarrow{\gamma_{[B]}} \emptyset$	Decay of BiP.
$\emptyset \xrightarrow{F_{[E_{1_m}]}} E_{1_m}$	Translation of Ero1 mRNA.
$E_{1_m} \xrightarrow{\gamma_{[E_{1_m}]}} \emptyset$	Decay of Ero1 mRNA.
$\emptyset \xrightarrow{\beta_{[E_1]}} E_1$	Translation of Ero1.
$E_1 \xrightarrow{\gamma_{[E_1]}} \emptyset$	Decay of Ero1.

$F_{[B_m]}$ - $F_{[B_m]} = \beta_{[B_m]}[1 + N_{[B_m]} f([H^s_{1_m}])]$ is a function that relates the transcription rate of BiP mRNA to the amount of spliced Hac1 ($H^s_{1_m}$). $\beta_{[B_m]} = .1625$ mol s^{-1} is the basal transcription rate (see Fitting section below). The function $f([H^s_{1_m}]) = [H^s_{1_m}]^2/(a_0 + a_1[H^s_{1_m}] + [H^s_{1_m}]^2)$ is the transcription hill function for the UPRE promoter where $a_0 = 296.5$ and $a_1 = 5.26$. The function is fitted (see Figure S8) to the normalized data in [*Credle et al.*(2005)] where the percent of spliced Hac1 mRNA is related to the LacZ reporter. $N_{[B_m]} = 4$ is used in our model and is within the range of values measured by the transcriptional reporter.

$\gamma_{[B_m]}$ - 6.67×10^{-4} s^{-1}. Decay rate of BiP mRNA corresponding to a 25 minute mean decay time.

$\beta_{[B]}$ - .25 s^{-1}. Translation rate of BiP.

$\gamma_{[B]}$ - 1.39×10^{-4} s^{-1}. Decay rate of BiP corresponding to a 2 hour mean decay time referenced from the modeling paper [*Axelsen and Sneppen*(2004)].

$F_{[E1_m]}$ - $F_{[E1_m]} = \beta_{[E1_m]}[1 + N_{[E1_m]} f([H^s_{1_m}])]$ is the transcription rate for Ero1 mRNA. $\beta_{[E1_m]} = 1.08$ mol s^{-1} is the basal transcription rate. The hill function $f([H^s_{1_m}])$ is the same as that used in $F_{[B_m]}$. $N_{[E1_m]} = 7$ is the value used in the model and is within the range of experimental measurements for the transcriptional reporter.

$\gamma_{[E1_m]}$ - 6.67×10^{-4} s^{-1}. Decay rate of Ero1 mRNA. Assumed to be same as that of BiP mRNA.

$\beta_{[E_1]}$ - .25 s^{-1}. Translation rate of Ero1. For more details see *Crucial Parameter Fitting* section below.

$\gamma_{[E_1]}$ - 1.39×10^{-4} s^{-1}. Decay rate of Ero1. Assumed to be same as that of BiP.

4 Kinetic Model Equations

Module 1: protein folding dynamics

$$
\begin{aligned}
\frac{d[U]}{dt} &= S_U - c_{[B]}[U][B] + \gamma_{[U\cdot B]}[U\cdot B] \\
&- c_{D_0} D_0[U] + c_{[E_1]}[E_1][U_d] + \gamma_B[U\cdot B] \\
\frac{d[U\cdot B]}{dt} &= c_{[B]}[U][B] - \gamma_{[U\cdot B]}[U\cdot B] \\
&- c_{D_0} D_0[U\cdot B] + c_{[E_1]}[E_1][U_d\cdot B] \\
&- \gamma_B[U\cdot B] - \gamma_{fold}[U\cdot B] \\
\frac{d[U_d]}{dt} &= -c_{[B]}[U_d][B] + \gamma_{[U\cdot B]}[U_d\cdot B] \\
&+ c_{D_0} D_0[U] - c_{[E_1]}[E_1][U_d] + \gamma_B[U_d\cdot B] \\
\frac{d[U_d\cdot B]}{dt} &= c_{[B]}[U_d][B] - \gamma_{[U\cdot B]}[U_d\cdot B] \\
&+ c_{D_0} D_0[U\cdot B] - c_{[E_1]}[E_1][U_d\cdot B] - \gamma_B[U_d\cdot B]
\end{aligned}
$$

Module 2: Ire1,Hac1 mRNA, and splicing reporter dynamics

$$\begin{aligned}
\frac{d[I_1]}{dt} &= -c_{[B]}[B][I_1] - c_{up}\Big([U] + [U_d] + [U_d \cdot B]\Big)[I_1] \\
&+ \gamma_{[I_1 \cdot B]}[I_1 \cdot B] + F_{[I_{1A}]}[I_{1A}] \\
\frac{d[I_1 \cdot B]}{dt} &= c_{[B]}[B][I_1] - \gamma_{[I_1 \cdot B]}[I_1 \cdot B] \\
\frac{d[I_{1A}]}{dt} &= c_{up}\Big([U] + [U_d] + [U_d \cdot B]\Big)[I_1] - F_{[I_{1A}]}[I_{1A}] \\
\frac{d[H^u_{1_m}]}{dt} &= \beta_{[H^u_{1_m}]} - \gamma_{[H^s_{1_m}]}[H^u_{1_m}] - \beta_{[H^s_{1_m}]}\min([I_{1A}], [H^u_{1_m}]) \\
\frac{d[H^s_{1_m}]}{dt} &= -\gamma_{[H^s_{1_m}]}[H^s_{1_m}] + \beta_{[H^s_{1_m}]}\min([I_{1A}], [H^u_{1_m}]) \\
\frac{d[R_{S_m}]}{dt} &= -\gamma_{[R_{S_m}]}[R_{S_m}] + \beta_{[H^s_{1_m}]}\min([I_{1A}], [H^u_{1_m}]) \\
\frac{d[R_S]}{dt} &= -\gamma_{[R_S]}[R_S] + \beta_{[R_S]}[R_{S_m}]
\end{aligned}$$

Module 3: BiP and Ero1 dynamics

$$\begin{aligned}
\frac{d[B_m]}{dt} &= \beta_{[B_m]}[1 + N_{[B_m]}]f([H^s_{1_m}])] - \gamma_{[B_m]}[B_m] \\
\frac{d[B]}{dt} &= \beta_{[B]}[B_m] - \gamma_{[B]}[B] - c_{[B]}[U][B] + \gamma_{[U \cdot B]}[U \cdot B] \\
&- c_{[B]}[U_d][B] + \gamma_{[U \cdot B]}[U \cdot B] + \gamma_{fold}[U \cdot B] \\
\frac{d[E_{1_m}]}{dt} &= \beta_{[E_{1_m}]}[1 + N_{[E_{1_m}]}f([H^s_{1_m}])] - \gamma_{[E_{1_m}]}[E_{1_m}] \\
\frac{d[E_1]}{dt} &= \beta_{[E_1]}[E_{1_m}] - \gamma_{[E_1]}[E_1]
\end{aligned}$$

To simulate the various mutants, the equations were adjusted as follows:

strain	alteration to the kinetic equations
wild type	none
hac1Δ	set $N_{[B_m]} = N_{[E_{1_m}]} = 0$, i.e. only basal transcription (no feedback).
ire1^{bipless}	set $c_{[I_1 \cdot B]} = \gamma_{[I_1 \cdot B]} = 0$.
hac1Δ + ire1^{bipless}	set $N_{[B_m]} = N_{[E_{1_m}]} = 0$. set $c_{[I_1 \cdot B]} = \gamma_{[I_1 \cdot B]} = 0$.

Starting from the equilibrium solution for non-stressed conditions (zero DTT), the DTT levels (represented by D_0 as the molecular number of DTT within the ER) were adjusted as a function of time, simultaneously solving the equations using the ordinary differential equation solver, ODE15s, in MATLAB.

4.1 Crucial Fitted Parameters

In the section, we discuss parameters for which we did not find any quantitative measurements or inferences in the literature. There are seven such parameters. To fit these parameters, we use a subset of the data describing the dose dependent splicing reporter experimental data sampled at 200 minutes after stress induction for the wild type, $hac1\Delta$, $ire1^{bipless}$, and $hac1\Delta$ + $ire1^{bipless}$. Our best fit is shown in Figure S9a. Figure S9b shows the results for the modeled washout experiment based on our fitted model predicting a delay in the ire1^{bipless} mutant, which was also experimentally verified. The details for parameter fitting are listed below.

S_u - The source rate of unfolding proteins, whose value is important relative to the value of total BiP. The best fitted results were obtained when the basal level of total BiP ($\approx 430,000$) was set to be 15-20 percent above the basal level of folding proteins. This value results in the population of free BiP to be around $60,000$. In the model, the larger the amount of free BiP to bound BiP, the slower the onset of splicing in the wild type system in the 5 mM DTT experiment. This is due to the fact free BiP sequesters Ire1 until there is enough unfolded proteins in the ER to titrate free BiP. Therefore, the choice of this parameter was set to match well with the onset of max splicing (20-30 minutes). However, in principle, this value can be changed without affecting the qualitative behavior of the system, especially the delay observed in the washout experiment. Instead, different values for S_u affect the *magnitude* of the delay. Figure S11a illustrates the sensitivity of the model to this parameter by varying the value of S_u. Here we plot the modeled washout experiment for $S_u = 370$ (dashed), 310 (solid), and 250 (dashed). All values produce a delayed response in the ire1^{bipless} case relative to the wild-type, but the delay dependent on the value of S_u.

E_{1_0} - The basal level of Ero1, $E_{1_0} = \beta_{[B_m]}\beta_{[B]}/(\gamma_{[B_m]}\gamma_{[B]})$, was fit to data from hac1Δ and hac1Δ+ ire1^{bipless}. This parameter was especially picked to fit the half maximal induction of the dose response curves for .4 and .25 mM DTT. Since these experiments have no transcriptional feedback, the basal level reaches approximately 1,350,000 molecules per ER volume, keeping in mind that Ero1 in this model represents the full system of molecules combating the effects of a similar number of DTT molecules.

$N_{[B_m]}$ - $N_{[B_m]} = 4$ was found to best fit the dose response data for the transcription feedback response of the wild type and ire1^{bipless} experiments. This value is within the range of the measured transcriptional reporters.

$N_{[E1_m]}$ - $N_{[E1_m]} = 7$ was found to best fit the dose response data for the transcription feedback response of the wild type and ire1^{bipless}. This value is within the range that transcriptional reporters measured.

The constants $c_{[I\cdot B]}$, $\gamma_{[I\cdot B]}$, c_{up} and $\gamma_{[I\cdot U]}$ dictate the system's dynamics, and determine the levels of free BiP and misfolded/unfolded proteins that can compete for free Ire1.

c_{up} - The value of c_{up} relative to $c_{[I\cdot B]}$ is very important. The competition between unfolded proteins and BiP for Ire1 is quantified by the association rate fluxes $c_{up}([U] + [U_d] + [U_d \cdot B])$ for the unfolded protein and $c_{[I\cdot B]}[B]$ for BiP. We found that the ratio $\alpha_{up} = c_{[I\cdot B]}/c_{up} = 150$ works best in fitting the model to data. This value makes intuitive sense since both Ire1 and the unfolded protein (even those with BiP attached) will generally diffuse much slower than

free BiP. Figure S11b shows that the quantitative aspects of the model are dependent on the variation to α_{up} for the values 100 (dashed), 150 (solid), and 250 (dotted). Here the intuition is that the lower the value of α_{up}, the lower the size of the relative population of misfolded proteins that is needed to compete for free Ire1.

$\gamma_{[I_1 \cdot B]}$ - $\gamma_{[I_1 \cdot B]}$ represents the dissociation rate of BiP from the inactive complex. This quantitiy is important since it determines the amount of time that an Ire1 molecule is free versus bound to BiP in a given condition of ER stress. Although no real quantitative measurements have been recorded, there are some estimates based on the work in [*Bertolotti et al.*(2000)] where BiP association to Ire1α in mammalian cells was studied. We will assume similar result for yeast. In the experiments in [*Bertolotti et al.*(2000)] for non-stressed cells, Ire1α/BiP complex and Ire1α were immunoprecipitated. It was found that the majority of Ire1α were in the complex form with BiP. Based on this, we can assume that at equilibrium, the ratio of the "ON" to "OFF" rates are the same as the ratio of the number of Ire1/BiP to free Ire1, neglecting active Ire1 which we'll assume to be zero for this calculation. Thus, we have $\frac{[I_1 \cdot B]}{[I_1]} = \frac{c_{[I_1 \cdot B]}[B]}{\gamma_{[I_1 \cdot B]}} = R$. In our model we set $R = 12.5$. Figure S11c plots the results of the washout experiments for 3 values of R: 125 (dashed), 12.5 (solid), 1.25 (dotted). In this sensitivity test, for a given value of R, we also scaled $\gamma_{[U \cdot B]}$ and $\gamma_{[I_{1A}]}$ by the relative change in R from its best fit value of 12.5, i.e 10 (a), 1 (b), .1 (c), respectively. The results in Figure S11c show that the system is robust for values of $R \geq 12.5$, but at $R = 1.25$, the full splicing response to 5 mM DTT does not occur. An explanation for this is that the basal level of active Ire1 (splicing) are higher in the non-stressed case for $R = 1.25$, thus are preadapted for higher levels of stress.

$\gamma_{[U \cdot B]}$ - There are no known measurements for $\gamma_{[U \cdot B]}$, the dissociation rate of BiP from folding complexes. Setting $\gamma_{[U \cdot B]}=\gamma_{[I_1 \cdot B]}$ worked well to fit the model.

$\gamma_{[I_{1A}]}$ - There are no known measurements for $\gamma_{[I_{1A}]}$, the deactivation rate constant of Ire1, where the general deactivation rate in our model is of the form $F_{[I_{1A}]} = \gamma_{[I_{1A}]} \frac{[I_0]^n}{[I_0]^n + [I_{1A}]^n}$ as represented in Figure S10b. The most important assumption of Ire1 deactivation that was crucial for fitting the model to the data was that the active state of Ire1 was stabilized as a function of increasing the active Ire1 population. For our model we were able to fit the data by setting $\gamma_{[I_{1A}]} = \gamma_{[I_1 \cdot B]}$, with a best fit of $n = 4.5, I_0 = 45$. Reasonable fits of the model were obtained over the ranges of $n = 3 - 6, I_0 = 20 - 60$. However, for $n = 1, I_0 = 15$, Figure S9c-d plots the results of the dose dependent splicing reporter sampled at 200 minutes as well the results for the washout experiment. Both the switch-like behavior the $ire1^{bipless}$ dose response and the delay in the $ire1^{bipless}$ washout experiment are lost. That said, we would like to point out that this cooperative function is a simplified representation of the ire1 cooperativity in the foci dynamics.

4.2 Additional Parameter Sensitivity Analysis

The modeling analysis implemented in the paper assumed the mean protein folding time, $1/\gamma_{fold}$, to be 20 minutes. However, the ratio S_u/γ_{fold} dictates the basal unfolded protein population. Therefore, we checked that the qualitative behavior of the model is insensitive to the folding time by varying the mean between 12 and 20 minutes, while keeping the ratio (basal population) constant. Figures S12a-b show the dose response curves at 200 minutes and the washout experiments, respectively, for folding times of 12, 15 and 20 (best fit) minutes. In all cases, the system exhibited the same

qualitative trends seen in the data. We also examined the sensitivity of the model to different values of the cytosolic diffusion coefficient, D_c, which affects many reaction constants simultaneously. We varied D_c over a range of $.5 < D_c < 2.0$ $\mu m^2 s^{-1}$. Over this range, the model robustly reproduced the trends seen in the data as seen in Figure S12c for the washout experiments. The dose response curves at 200 minutes (not shown) were all very similar as well.

Our model assumes an Ire1 molecular count of 256 molecules/cell [*Ghaemmaghami et al.* (2003)], which is based on an average value over many cells. We varied the range of Ire1 from 232 to 281. A notable feature of the model is that while the deactivation dynamics of the wild type system are still faster than the $ire1^{bipless}$ mutant in the simulation of the washout experiment, a large variability in the shutoff delay was was observed as the number of Ire1 molecules varied for the $ire1^{bipless}$ mutant (Figure S12d). This observation constitutes an interesting experimental prediction.

References

[*Axelsen and Sneppen*(2004)] Axelsen J., Sneppen, K. , (2004) Quantifying the benefits of translation regulation in the unfolded protein response. *Physical Biol.*, 1:159-65.

[*Berg and Purcell*(1977)] Berg H., Purcell, W., (1977) Physics of Chemoreception. *Biophys. Journ.*, 20:193-219.

[*Bertolotti et al.*(2000)] Bertolotti A., Zhang, Y., Hendershot, L., Harding, H., Ron, D., (2000) Dynamic interaction of BiP and ER stress transducers in the unfolded protein response. *Nat. Cell Biol.*, 2(6):326-32.

[*Credle et al.*(2005)] Credle J., Finer-Moore, J., Papa, F., Stroud, R., Walter, P., (2005) On the mechanism of sensing unfolded protein in the endoplasmic reticulum. *Proc. Natl. Acad. Sci. USA*, 102(52):18773-84.

[*Ghaemmaghami et al.* (2003)] Ghaemmaghami S, Huh W-K, Bower K, Howson RW, Belle A, Dephoure N, O'Shea EK, Weissman JS. (2003) Global analysis of protein expression in yeast. *Nature*, 425:737-41.

[*Hebert and Molinari*(2007)] Hebert, D. and Molinari, M. (2007) In and Out of the ER: Protein Folding, Quality Control, Degredation, and Related Human Diseases. *Physiol Rev*, 87:1377-1408.

[*Mori et al.*(1997)] Mori K., Kawahara, T., Hideki, Y., Takashi, Y., (2003) Endoplasmic Reticulum Stress-induced mRNA Splicing Permits Synthesis of Transcription Factor Hac1p/Ern4p That Activates the Unfolded Protein Respsonse. *Mol. Biol. Cell*, 8:1845-62.

[*Sidrauski et al.*(1996)] Sidrauski C., Cox, J., Walter, P., (1996) tRNA Ligase is Required for Regulated mRNA Splicing in the Unfolded Protein Response. *Cell*, 87:405-413.

[*Walter*] Walter, P., Personal Communication.
